# Global burden and trends analysis of common cancers attributable to dietary risks from 1990 to 2021 and projection to 2050: a secondary analysis for the global burden of disease study 2021

**DOI:** 10.3389/fnut.2025.1673422

**Published:** 2025-12-19

**Authors:** Tingting Wei, Yunhai Li, Ze Zhang, Yijing Xu, Hongbo Huang, Ying Huang, Jiaying Li, Zheng Gong, Zhiqi Hu, Yichen Wang, Aijie Zhang, Fan Li

**Affiliations:** 1 Department of Breast and Thyroid Surgery, The First Affiliated Hospital of Chongqing Medical University, Chongqing, China; 2 Health Management Center of University Town Hospital Affiliated to Chongqing Medical University, Chongqing, China

**Keywords:** neoplasms, global burden of disease study, dietary risk factors, sociodemographic index, health inequality

## Abstract

**Background:**

Dietary risk factors play crucial roles in the carcinogenesis and progression of cancer. However, the global burden of diet-related neoplasms (DRNs) remains underexplored.

**Methods:**

Age-standardized disability-adjusted life years (DALYs) and mortality rates for DRNs were extracted from the Global Burden of Disease (GBD) Study 2021. These were categorized by dietary risk, sex, age, and sociodemographic index (SDI) level. Predictions of DALY and mortality trends from 2021 to 2050 were generated via the Bayesian age-period-cohort models. Cross-country inequalities were assessed using the slope index of inequality and the concentration index.

**Results:**

In 2021, dietary risk contributed to 16.40 million DALYs and 0.67 million deaths from DRNs, including esophageal, stomach, colon and rectum, tracheal, bronchus, and lung, breast, and prostate cancers. Diets high in red meat and low in whole grains were primary dietary risks for DRNs. Globally, the age-standardized DALY and mortality rates related to DRNs have decreased over the past three decades and are projected to continue to decline until 2050. The DRNs burden is particularly heavy for colon and rectal cancer patients, patients in high and high-middle SDI regions, men, and those aged ≥70 years. Cross-country inequalities reveal that the slope index of inequality rose for DRNs from 262.74 in 1990 to 296.14 in 2021. The concentration index was 0.27 in 1990 and 0.30 in 2021.

**Conclusion:**

This study revealed significant variations in the burden of DRNs across different age groups, sexes, regions, and countries, emphasizing the potential for tailored cancer screening strategies targeting populations with higher risks of DRNs.

## Introduction

Cancer represents a significant public health challenge, with an estimated 35 million new cases projected worldwide by 2050 (1). Although various risk factors contribute to cancer development, a suboptimal diet is responsible for more deaths globally than any other risk factor (2, 3). Robust evidence from a systematic review indicated that consuming fruits and vegetables has a protective effect against colorectal, breast, and lung cancers (2). Higher intakes of calcium, dairy, and whole grains and lower consumption of red and processed meat were related to a reduced risk of colorectal cancer (CRC) (2, 4). Conversely, an increased risk of prostate cancer (PC) has been associated with higher intakes of milk and dietary calcium (5). High salt intake and low fruit consumption are also potential risk factors for stomach cancer (SC) (6). The World Cancer Research Fund (WCRF) Third Expert Report concludes that diet is a modifiable cancer risk factor (4), with approximately one in five cancers can be prevented by a healthy diet globally (7). Thus, optimizing dietary intake may be a cost-effective strategy for cancer prevention (8).

Despite decades of epidemiological research, scientific evidence regarding the effects of many specific foods on cancer remains inconsistent or insufficient (2). Key limitations include insufficient geographically representative data on dietary consumption, inaccurate characterization of the population distribution of dietary intake, and inadequate accounting for biases from different sources of dietary assessment (3). Although GBD-based studies have provided valuable insights, most focus on individual cancers or single dietary risk factors, and few have analyzed diet-related neoplasms (DRNs) as an integrated group. Consequently, the overall burden, long-term trends, and global or regional inequalities associated with DRNs remain poorly characterized. DRNs share common, modifiable dietary etiologies, and evaluating them as a group provides a clearer picture of the overall cancer burden attributable to diet. This integrated approach also helps identify population disparities and guide targeted dietary interventions. Sustainable Development Goals (SDGs) target 3.4 aims to reduce global premature mortality by one-third for noncommunicable diseases (e.g., cancer) by 2030 (9). Thus, accurate and region-specific estimates of DRNs burden are crucial for guiding global health policy and resource allocation.

To our knowledge, the Global Burden of Diseases (GBD) is the only study comprehensively quantifying cancer burden attributable to a broad set of modifiable risk factors (10). GBD 2021 provides the latest information on the global burden of cancer attributable to risk factors. In this study, we extracted and analyzed data from the GBD 2021 to assess the mortality and disability-adjusted life years (DALYs) of common neoplasms attributed to dietary risk factors, categorized by sex, age, and sociodemographic index (SDI) from 1990 to 2021 globally. We also predicted the DALYs and mortality rates up to 2050. Furthermore, we conducted a cross-country inequality analysis to examine whether there are SDI level-related inequalities in the burden of these diet-related neoplasms (DRNs) across countries and to determine the magnitude and trends over time. The results can help policymakers implement preventive measures and tackle the growing DRNs burden.

## Materials and methods

### Data sources

The data used in this study were derived from the GBD 2021, which is freely available from the Institute for Health Metrics and Evaluation (IHME, https://vizhub.healthdata.org/gbd-results/). We utilized the estimated numbers of DALYs and mortality, age-standardized rates of DALYs (ASDRs), and age-standardized mortality rates (ASMRs) of common neoplasms attributable to dietary risks across 204 countries, 21 GDB regions, and 5 SDI regions. The global population estimates (up to 2100) were obtained from the GBD database.^1^ The use of anonymized, publicly available epidemiologic data did not require ethical approval, and patient informed consent forms were not necessary when accessing and downloading the data from the database.

### Definitions and dietary risk factors

#### ICD codes for the DRNs

The DRNs referred to in this study were coded according to the International Classification of Diseases 10th revision (ICD-10) as follows: C15–C15.9 for esophageal cancer (EC); C16–C16.9 for stomach cancer (SC); C18–C19.0, C20, and C21–C21.8 for colon and rectal cancer (CRC); C33 and C34–C34.92 for tracheal, bronchus, and lung cancer (TBLC); C50–C50.629 and C50.8–C50.929 for breast cancer (BC); and C61–C61.9 for prostate cancer (PC).

#### Disability-adjusted life years (DALYs), sociodemographic index (SDI) and population-attributable fraction (PAF)

DALYs in the GBD database refer to the sum of years lost due to premature death (YLLs) and years lived with disability (YLDs), which are also defined as years of healthy life lost. Previous studies have described the estimation process in detail (11, 12). The socioeconomic development status was indexed by the SDI, which consists of the total fertility rate in women aged <25 years, average years of schooling (aged >15 years), and lag-distributed income per person, ranging from 0 to 1 (13). The GBD database classified regions and countries according to their SDI values to determine whether disease burdens varied with SDI values (11). 204 countries and territories were classified under 5 SDI levels: low SDI (<0.45), low-middle SDI (0.45 to 0.61), middle SDI (0.61 to 0.69), high-middle SDI (0.69 to 0.80), and high SDI (≥0.80) (11). The PAF, which corresponds to the “percent” metric in the GBD database, is estimated independently for each risk factor and represents the proportion of a disease that could have been prevented if past exposure to the risk factor had been reduced to the theoretical minimum risk exposure level (TMREL) (14). It quantifies the attributable burden of cancer associated with dietary risk factors.

#### Selection of dietary risk factors

The GBD 2021 includes a total of 88 risk factors broadly categorized into three groups: (1) environmental and occupational, (2) behavioral, and (3) metabolic (10). It systematically evaluates the associations between these risk factors and 155 health outcomes, ultimately analyzing 631 risk–outcome pairs (15). These assessments adhere to the WCRF criteria for convincing or likely causal evidence. When integrating risk–outcome pairs, the GBD study draws upon data from original randomized controlled trials (RCTs), cohort studies, pooled cohort studies, and case–control studies. To ensure robust causal inferences, it employs the Burden of Proof Risk Function and a star rating system, which enhance data reliability, prioritize conservative risk estimations, and minimize potential biases (15). Within the GBD Comparative Risk Assessment framework, 15 dietary risk factors are considered. Of these, nine have been identified as relevant to the development of diet-related neoplasms (DRNs) based on their established risk–outcome associations. These nine dietary factors include a diet low in whole grains, milk, calcium, fruits, vegetables, and fiber, and a high intake of processed meat, red meat, and sodium. Diets low in whole grains are defined as consuming less than 160–210 g/day of whole grains (bran, germ, and endosperm in their natural proportion) from breakfast cereals, bread, rice, pasta, biscuits, muffins, tortillas, pancakes, and other sources. Diets low in milk represent an average daily consumption of less than 280–340 g/day for males and 500–610 g/day for females of milk, including non-fat, low-fat, and full-fat milk, excluding soy milk and other plant derivatives. Diets low in calcium represent an average daily consumption of less than 0.72–0.86 g/day for males and 1.06–1.20 g/day for females of calcium from all sources, including milk, yogurt, and cheese. Diets low in fruit represent an average daily consumption of less than 340–350 g/day of fruit, including fresh, frozen, cooked, canned, or dried fruit, excluding fruit juices and salted or pickled fruits. Diets high in red meat represent any intake of red meat, including beef, pork, lamb, and goat but excluding poultry, fish, eggs, and all processed meats. Diets high in sodium represent an average daily consumption of more than 5 g/day of sodium. Diets high in processed meat represent any intake of meat preserved by curing, smoking, salting, or the addition of chemical preservatives. Diets low in vegetables represent an average daily consumption of less than 306–372 g/day of vegetables, including fresh, frozen, cooked, canned, or dried vegetables and excluding legumes and salted or pickled vegetables, juices, nuts and seeds, and starchy vegetables such as potatoes or corn. Diets low in fiber represent an average daily consumption of less than 22–25 g/day of fiber from all sources, including fruits, vegetables, grains, legumes, and pulses (Supplementary Table S1). The details of the definitions and quantifying methodology of the risk attribution are described elsewhere (15).

### Statistical analysis

To reflect trends in the burden of DRNs over the past 30 years, we calculated the estimated annual percentage changes (EAPCs) and their 95% confidence intervals (CIs) in ASDRs and ASMRs for DRNs from 1990 to 2021 by age group (15–49 years, 50–69 years, 70 + years), sex (male, female), and SDI (low SDI, low-middle SDI, middle SDI, high-middle SDI, and high SDI). The calculation method has been detailed in a previous study (16). Changes in each outcome were calculated by comparing the data for 2021 with those for 1990. If the EAPCs and the lower limits of the CI were positive, the ASR tended to increase. In contrast, if the EAPCs and the upper limits were negative, the ASR tended to decrease (17). To predict the ASDRs and ASMRs of DRNs and their 95% CIs from 2021 to 2050, the Bayesian age-period-cohort (BAPC) and Integrated nested Laplace approximation (INLA) packages of R software were used for analysis. In this framework, the observed events were assumed to follow a Poisson distribution, and age, period, and cohort effects were assigned second-order random walk (RW2) priors. For the precision parameters of the RW2 components and the intercept, we used the weakly informative priors recommended by the BAPC package. Posterior distributions and inference were obtained via Integrated Nested Laplace Approximation (INLA) (18). This approach allows for smooth temporal trends while appropriately accounting for age, period, and cohort effects. Compared with alternative methods such as ARIMA or joinpoint regression, BAPC provides probabilistic forecasts with credible intervals, which are essential for quantifying predictive uncertainty (19). This GBD forecast was primarily based on the Global Population Forecasts 2017–2100 data and age-standardized DALY and death data for DRNs from 1990 to 2021. The distributive inequality of the DRNs burden across countries was measured by the slope index of inequality (SII) and concentration index, representing absolute and relative gradient inequality, respectively (20). The SII was calculated by regressing national DALY rates in all-age populations on an SDI-associated relative position scale, defined by the midpoint of the cumulative range of the population ranked by the SDI. Heteroskedasticity was accounted for by the use of a weighted regression model. The concentration index was derived by numerically integrating the area under the Lorenz concentration curve, which was fitted using the cumulative fraction of DALYs and the cumulative relative distribution of the population ranked by the SDI (21). All of the statistical analyses were performed using R software (version 4.3.0) and GraphPad Prism version 8.0.1 (GraphPad Software Inc., La Jolla, CA, USA).

## Results

### Dietary risk contributes to the burden of all causes or neoplasms

From 1990 to 2021, across 21 GBD regions and 204 countries and territories, a total of 4704.82 (1333.40–6818.01) million DALYs and 188.32 (53.59–278.35) million deaths were attributable to dietary risk factors, as recorded in the GBD 2021 study. Among these, 444.98 (128.76–817.87) million DALYs and 17.47 (5.18–31.85) million deaths were specifically linked to DRNs. Within DRNs, males accounted for 52.0% [231.37 (72.71–441.10) million] DALYs and 50.9% [8.90 (2.86–16.87) million] deaths, while females accounted for 213.62 (56.09–384.11) million DALYs and 8.57 (2.37–15.23) million deaths. The changes in dietary risk rankings from 1990 to 2021 for ASDRs due to diet-related all causes or neoplasms are summarized in Supplementary Figure S1. Despite significant declines in ASDRs from 1990 to 2021, the top four dietary risk factors (including diets low in fruit, whole grains, and vegetables and high in sodium) contributing to global all-cause burden remained unchanged. The most notable change was attributable to high sweetened beverages, with a 69.9% global decrease in ASDRs (Supplementary Figure S1A). Globally, diets high in red meat (ASDR: 85.70 in 1990 and 69.56 in 2021) and low in whole grains (ASDR: 63.47 in 1990 and 50.19 in 2021) and milk (ASDR: 49.31 in 1991 and 40.40 in 2021) have been the top three dietary risk factors for DRN DALYs burden in both 1990 and 2021 (Supplementary Figure S1B; Supplementary material S2). Although the ranks of ASDRs increased due to low calcium and fruit intake, the burden of DRNs attributed to these risk factors declined significantly (Supplementary Figure S1B).

**Figure 1:**
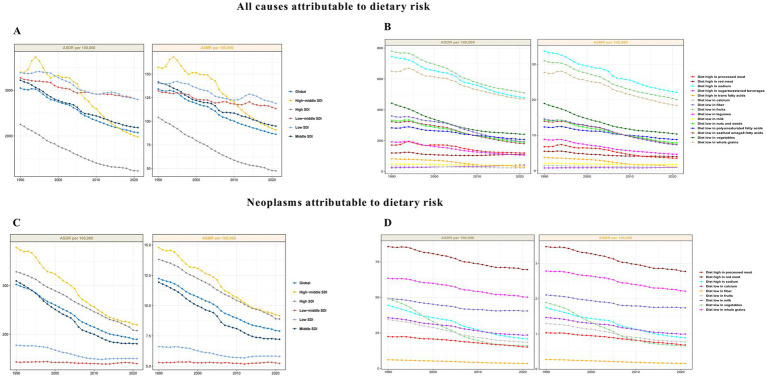
Age-standardized rate (ASR) for dietary risks attributable to all causes according to global burden. **(A)** ASR for dietary risks attributable to all causes in both sexes. **(B)** Different dietary risks attributed to all causes for ASR in both sexes. **(C)** ASR for dietary risks attributed to neoplasms in both sexes. **(D)** Different dietary risks attributed to neoplasms for ASR in both sexes.

In 2021, dietary risk factors were responsible for an estimated 178.26 million (49.77–260.91) DALYs globally, with a PAF of 6.19% (1.76–9.14). Among these, a diet low in fruits was the leading contributor, accounting for 24.6% of diet-related DALYs, equivalent to 43.78 million (17.67–65.16) DALYs (Supplementary Table S2). Deaths associated with dietary risk factors were 7.21 (1.96–10.77) million with a PAF of 10.63% (2.90–15.94) (Supplementary Table S2). From 1990 to 2021, both DALYs and deaths from all causes attributable to dietary risk factors steadily declined, with high SDI regions consistently showing the lowest ASDRs and ASMRs (Figure 1A). Although diets high in sodium and low in fruits and whole grains had the highest all-cause ASDRs and ASMRs, a notable downward trend was observed from 1990 to 2021 (Figure 1B). Regarding neoplasms, dietary risk factors contributed to 16.40 (4.94–29.03) million DALYs and 0.67 (0.21–1.18) million deaths in 2021, accounting for 9.2% of total DALYs and 9.3% of total deaths, respectively. The PAF for dietary risk factors was 6.47% (2.01–11.53) for DALYs and 6.77% (2.20–12.05) for deaths (Supplementary Table S2). From 1990 to 2021, the contribution of dietary risks to cancer ASDRs and ASMRs has also declined globally. The high and high-middle SDI quintiles had higher ASDRs and ASMRs than the low and low-middle SDI quintiles, but these rates declined more rapidly in the higher SDI quintiles (Figure 1C). The impact of each dietary risk factor on the burden of DRNs has decreased, with a low vegetable diet showing the most significant reduction over the past 30 years (Figure 1D).

### Regional distribution of the DRNs burden

**Figure 2:**
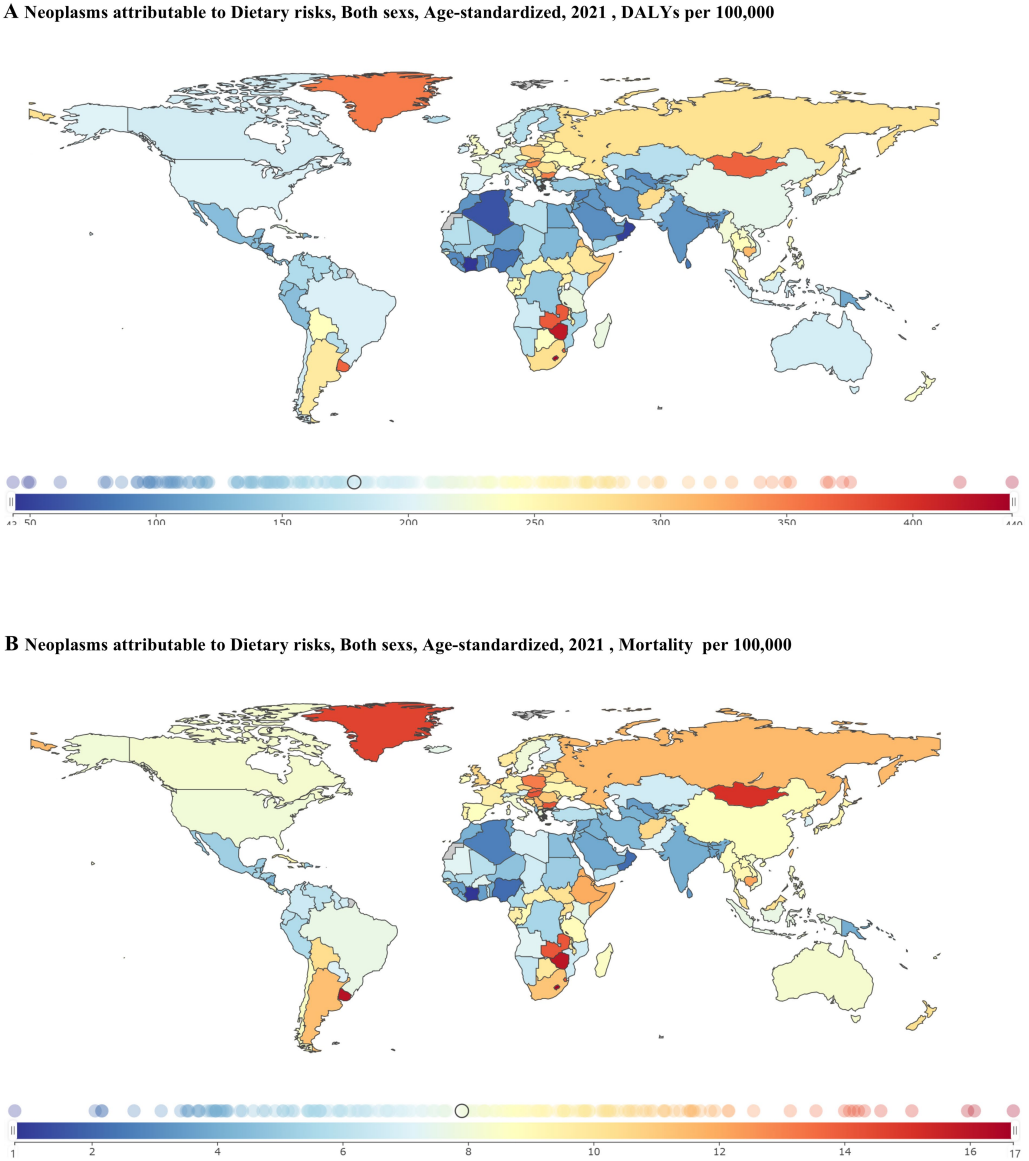
ASDR **(A)** and ASMR **(B)** for dietary risk-related neoplasms in 204 countries and territories, both sexes, in 2021. ASDR, age-standardized DALY rates; ASMR, age-standardized mortality rates.

In 2021, there was a significant geographical bias in the DRNs burden (Figure 2; Supplementary Figure S2; Supplementary Tables S3–S4). Central Europe [282.90 (73.39–477.40) DALYs and 12.13 (3.28–20.29) deaths per 100,000 population], Southern Latin America [251.76 (64.38–434.85) DALYs and 10.84 (2.91–18.68) deaths per 100,000 population], and Southern Sub-Saharan Africa [297.88 (83.75–492.82) DALYs and 11.74 (3.52–19.17) deaths per 100,000 population] had a higher burden of DRNs, whereas South Asia [112.31 (36.66–193.32) DALYs and 4.21 (1.48–7.20) deaths per 100,000 population] and Western Sub-Saharan Africa [103.80 (34.10–183.58) DALYs and 3.53 (0.69–6.27) deaths per 100,000 population] experienced a relatively lighter burden (Figure 2; Supplementary Figure S2; Supplementary Table S3). Among 204 countries, Lesotho had the highest ASDRs [439.34 (129.18–748.79) DALYs per 100,000 population] and ASMRs [16.69 (5.28–28.00) mortality per 100,000 population] for DRNs (Figure 2; Supplementary Table S4). In contrast, Côte d’Ivoire had the lowest ASDRs [43.26 (−38.15 to 89.50) DALYs per 100,000 population] and ASMRs [0.77 (−3.45 to 3.18) deaths per 100,000 population] for DRNs (Figure 2; Supplementary Table S4).

With respect to each DRN, globally, 9.46 million DALYs (57.6% of total DRNs DALYs) and 0.41 million deaths (60.6% of total DRNs deaths) were due to CRC in 2021, followed by BC, SC, TBLC, and EC. Among all DRNs, CRC had the highest PAF for dietary risks, with 38.74% (13.49–58.50) for DALYs and 38.87% (13.38–58.80) for deaths (Supplementary Table S2). In most regions, CRC had the highest ASDRs and ASMRs attributable to dietary risks, followed by BC in 2021 (Supplementary Figure S2). In 2021, diets high in red meat and low in whole grains were the primary dietary factors contributing to the burden of DRNs in 16 out of the 21 GBD regions (Supplementary Figure S2). In African regions, the leading risks have shifted to diets low in vegetables and high in red meat, especially in Central Sub-Saharan Africa, where diet low in vegetables became the primary dietary risk factor. In Southern Latin America, Europe, Australasia, and High-income North America, the impact of a diet high in processed meat was notable (Supplementary Figure S2).

From 1990 to 2021, the DRNs burden attributable to dietary risk factors declined in most regions, but increased in Southern Sub-Saharan Africa (EAPC in ASDR: 0.44; EAPC in ASMR: 0.49) and Western Sub-Saharan Africa (EAPC in ASDR: 0.77; EAPC in ASMR: 0.79) (Supplementary Figure S3). Among these risk factors, diets low in sodium, calcium, fruits, and low consumption of vegetables were the major factors contributing to the decline of DRNs burden in these regions (Supplementary Figure S3). The most dramatic decrease in DRNS burden was ascribed to low consumption of vegetables in high-middle SDI regions (EAPC in ASDR: −7.48; EAPC in ASMR: −6.91), followed by countries with middle SDI. Conversely, countries with middle SDI showed a sharp increase in DRNs burden, which is attributable to diets high in processed meat (EAPC in ASDR: 1.30; EAPC in ASMR: 1.22) (Supplementary Figure S3).

### The impact of individual dietary risk on DRNs outcomes

**Figure 3:**
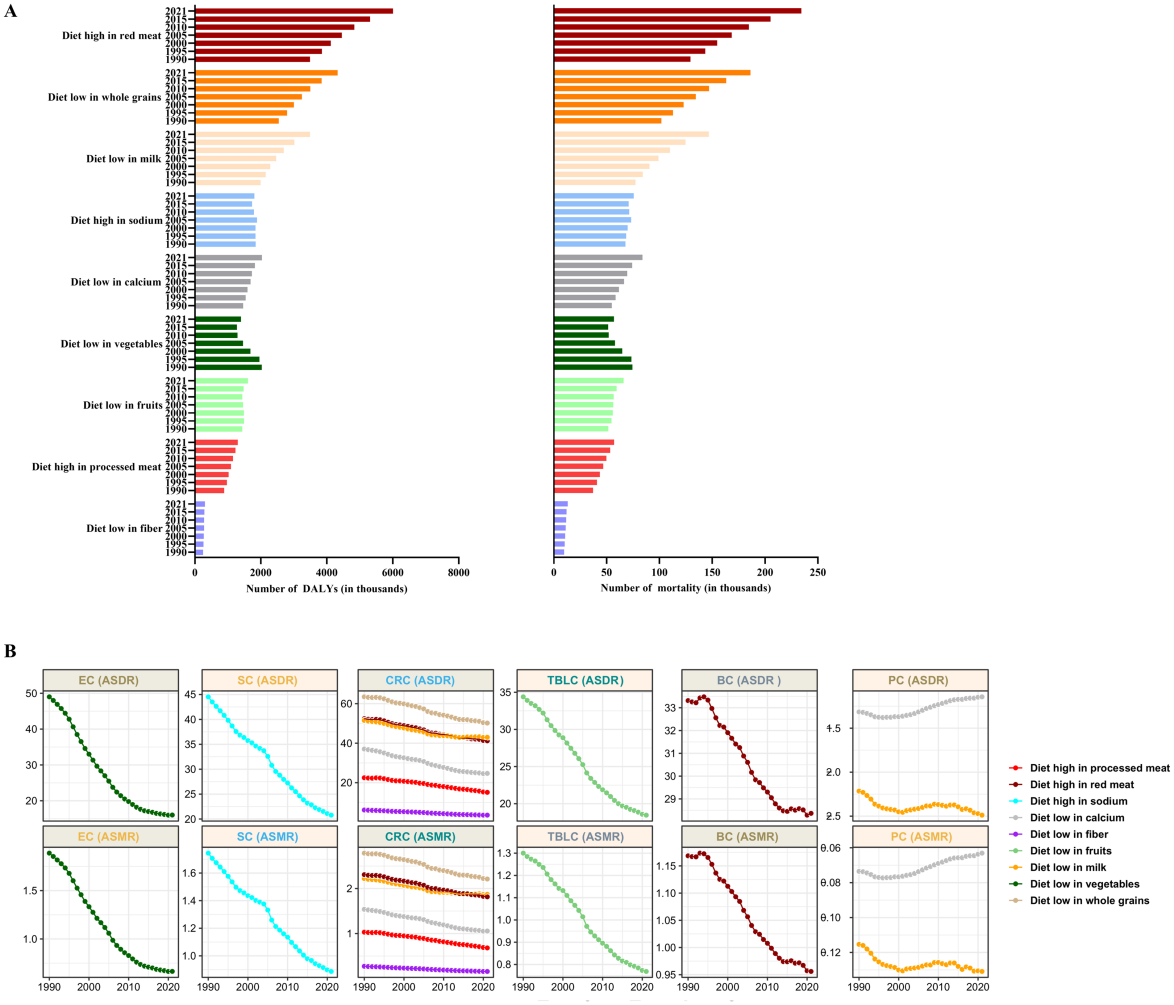
DALYs and mortality for the global burden of neoplasms according to differences of dietary risks from 1990 to 2021. **(A)** Diet-related neoplasms DALYs and deaths. **(B)** Different dietary risks attributed global ASDR and ASMR of common cancers in both sexes. ASDR, age standardized DALYs rate; ASMR, age standardized mortality rate.

In 2021, diets high in red meat (6.00 million DALYs; 0.23 million deaths) and low intake of whole grains (4.33 million DALYs; 0.19 million deaths) were the top two dietary risk factors contributing to the global DRNs burden, with a PAF of 2.37 and 1.71% for DALYs and 2.37 and 1.88% for deaths, respectively (Supplementary Table S2). Over the past three decades, the number of DRNs attributed to diets high in red meat, low in whole grains, low in milk, low in calcium, and high in processed meat has gradually increased, whereas the number of DRNs attributed to low vegetable intake has shown a declining trend (Figure 3A). From 1990 to 2021, six out of nine dietary risks had a significant impact on CRC burden, including diets low in whole grains, low in milk, high in red meat, low in calcium, high in processed meat, and low in fiber, while the other DRNs typically have one or two associated dietary risk factors (Figure 3B). Notably, low intakes of milk and calcium were negatively associated with PC. In addition to PC, the contribution of dietary risk to common DRNs notably decreased from 1990 to 2021 (Figure 3B).

### DRNs stratified by SDI

**Figure 4:**
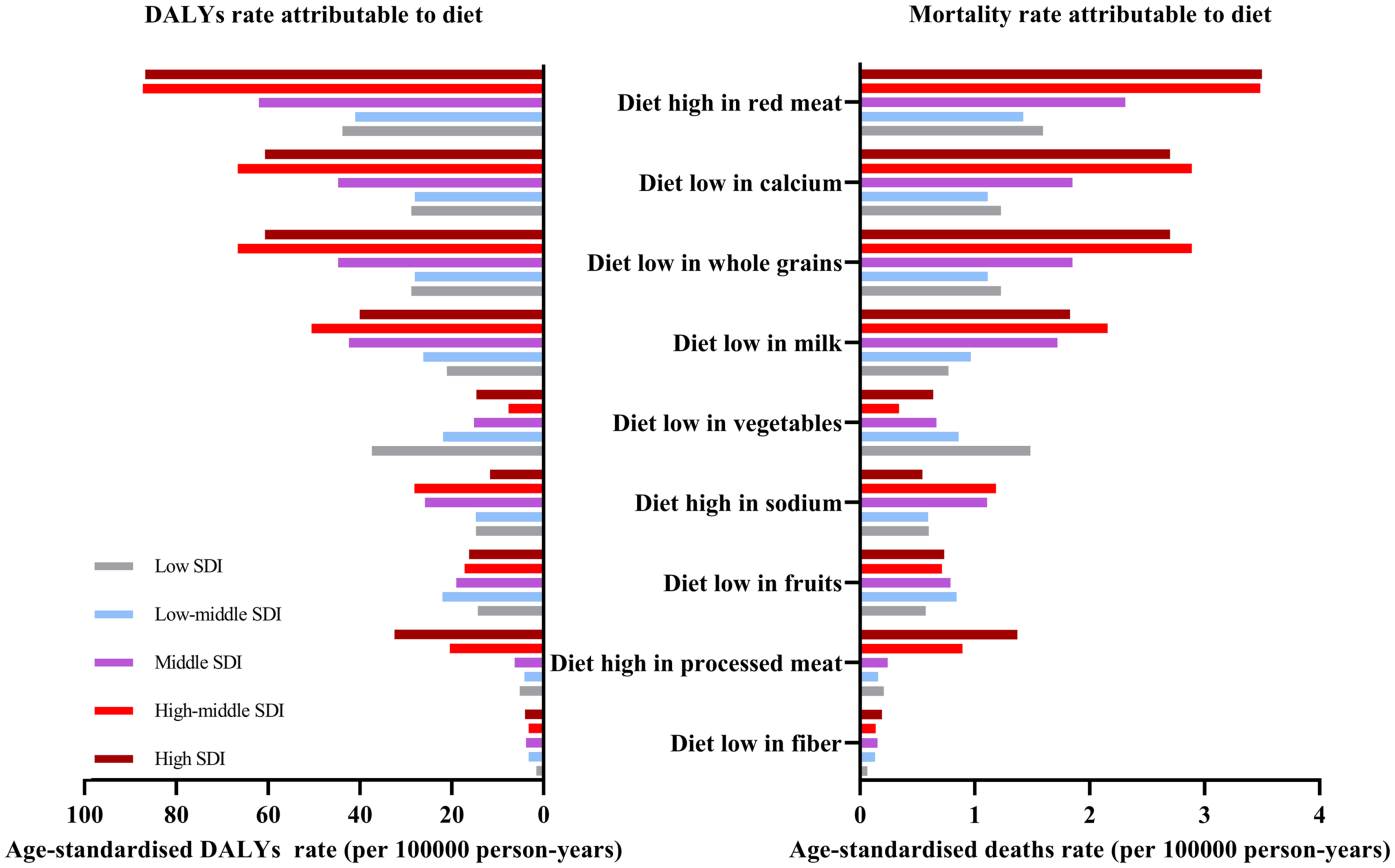
ASDR and ASMR attributable to individual dietary risks at the SDI level in 2021. ASDR, age-standardized DALYs rates; ASMR, age-standardized mortality rates; SDI, sociodemographic index.

High, high-middle, and middle SDI countries were at the greatest risk of DALYs and deaths from high consumption of red meat in 2021 (Figure 4). Compared with regions with higher SDIs, regions with lower SDIs have a greater risk of DRNs burden attributable to a diet low in vegetables (Figure 4). Over the past 30 years, except for PC, the ASDRs and ASMRs of DRNs have decreased more significantly in the high-middle and middle SDI regions than in the low-middle and low SDI regions (Supplementary Figure S4A). Furthermore, stratified by SDI, both ASDRs and ASMRs for dietary-attributed CRC and BC increased with higher SDI ranks, while the burden of diet-related EC was the lowest in high and high-middle SDI regions (Supplementary Figure S4A). Across the five types of SDI regions, an increase in EAPC for BC and PC attributed to dietary risks was observed in low and low-middle SDI regions from 1990 to 2021 (Supplementary Figure S4B). In contrast, the most significant decline in ASDRs and ASMRs of diet-related EC and TBLC was observed in the middle and high-middle SDI regions (Supplementary Figure S4B).

### DRNs stratified by gender and age groups

**Figure 5:**
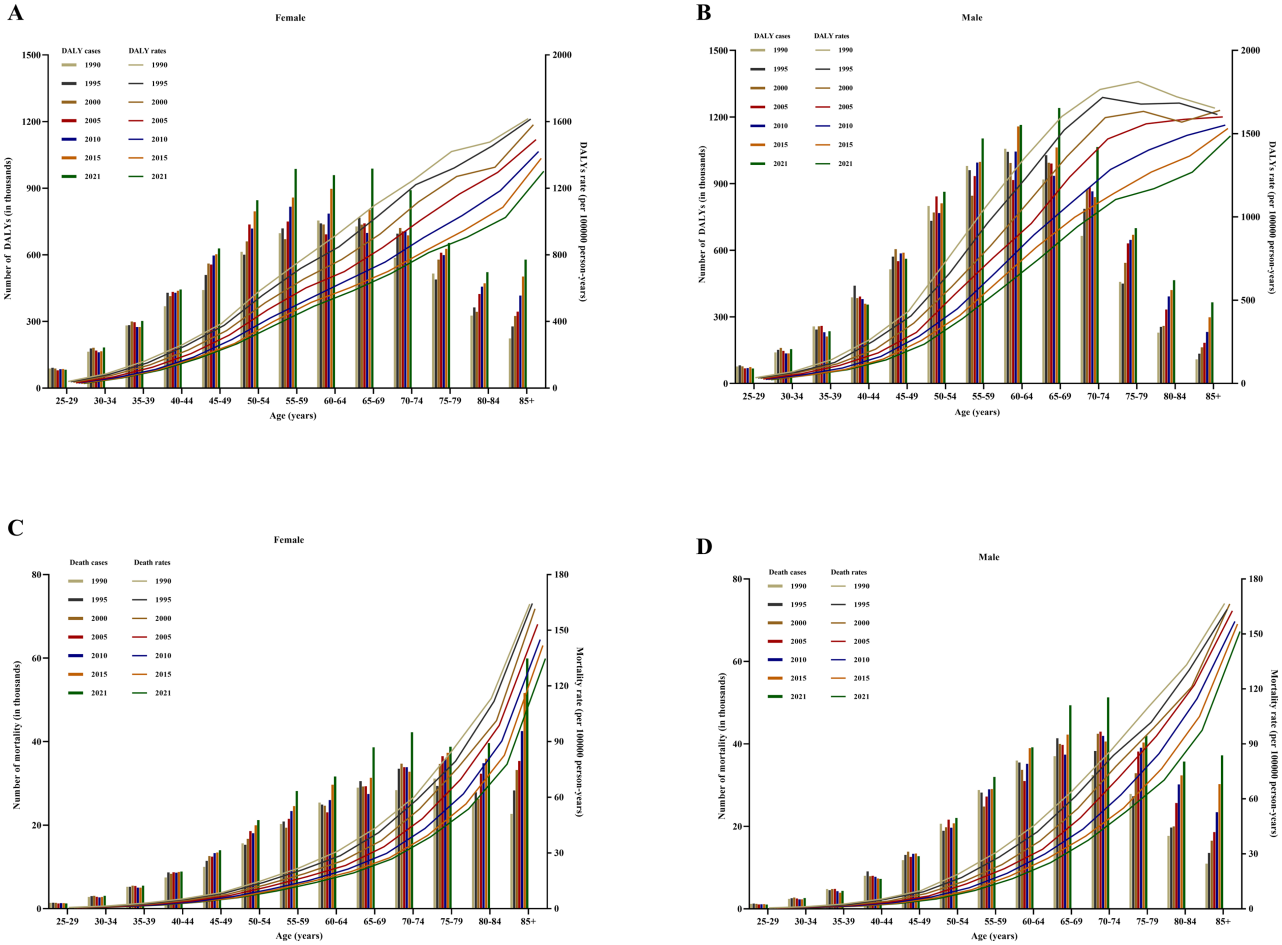
Age- and gender-specific DALYs and mortality of DRNs (bars) and share of diet-related on ASDR and ASMR (lines) from 1990 to 2021 in global. **(A)** DALYs of DRNs in female. **(B)** DALYs of DRNs in male. **(C)** Mortality of DRNs in female. **(D)** Mortality of DRNs in male. DRNs, diet-related neoplasms; ASDR, age-standardized DALYs rates; ASMR, age-standardized mortality rates; SDI, sociodemographic index.

In 2021, a significant sex bias was observed in the burden of common DRNs globally (Figure 5; Supplementary Figure S5; Supplementary material S2). The DALYs and death rate of DRNs were 203.61 (70.43–369.27) DALYs per 100,000 population and 8.73 (3.05–15.83) deaths per 100,000 population in men and 177.90 (46.76–316.47) DALYs per 100,000 population and 7.21 (2.04–12.52) deaths per 100,000 population in women, respectively (Supplementary material S2). Dietary risks led to the highest ASDRs [130.73 (41.56–203.42) DALYs per 100,000 population in men; 90.96 (33.62–134.74) DALYs per 100,000 population in women] and ASMRs [5.74 (1.81–8.94) deaths per 100,000 population in men; 4.06 (1.47–6.04) deaths per 100,000 population in women] of DRNs for CRC for both genders in 2021 (Supplementary Figure S5A; Supplementary material S2). From 1990 to 2021, except for PC, both sexes experienced a decline in DALYs and mortality rates for DRNs (Supplementary Figure S5A). Furthermore, except for BC, men consistently had higher ASDRs and ASMRs of DRNs than women over the past three decades (Supplementary Figure S5A). The decline in the EAPC for the ASDRs and ASMRs of EC, SC, CRC, and TBLC was pronounced in females. In contrast, male BC showed a slight upward trend in the ASDRs (Supplementary Figure S5B).

The number of DRN DALYs and deaths attributable to dietary risk factors varied by age group in 2021 (Figure 5; Supplementary material S2). DALYs peaked in both genders aged 65–69 years (1.24 million DALYs in men; 0.99 million DALYs in women), while deaths peaked in males aged 70–74 years (0.05 million deaths) and increased with age in females, peaking at 85+ years (0.06 million deaths) (Figure 5; Supplementary material S2). Despite an increase in the absolute number of DALYs and deaths from 1990 to 2021, the ASDRs and ASMRs for DRNs in specific age groups have decreased (Figure 5). As shown in Supplementary Figure S6A, those over 70 years old have shown significantly higher DALY and death rates from dietary risks from 1990 to 2021. In 2021, the highest DALY and mortality rate attributable to dietary risks were observed for CRC in patients aged over 70 years (696.98 DALYs per 100,000 population; 46.61 deaths per 100,000 population) (Supplementary Figure S6A; Supplementary material S2). We analyzed the percentage changes in ASDRs and ASMRs for each age group from 1990 to 2021 (Supplementary Figure S6B). Except for PC, DALY and mortality rates for DRNs showed a decreasing trend across all age groups.

### Future forecasts of global DRNs burden to 2050

**Figure 6:**
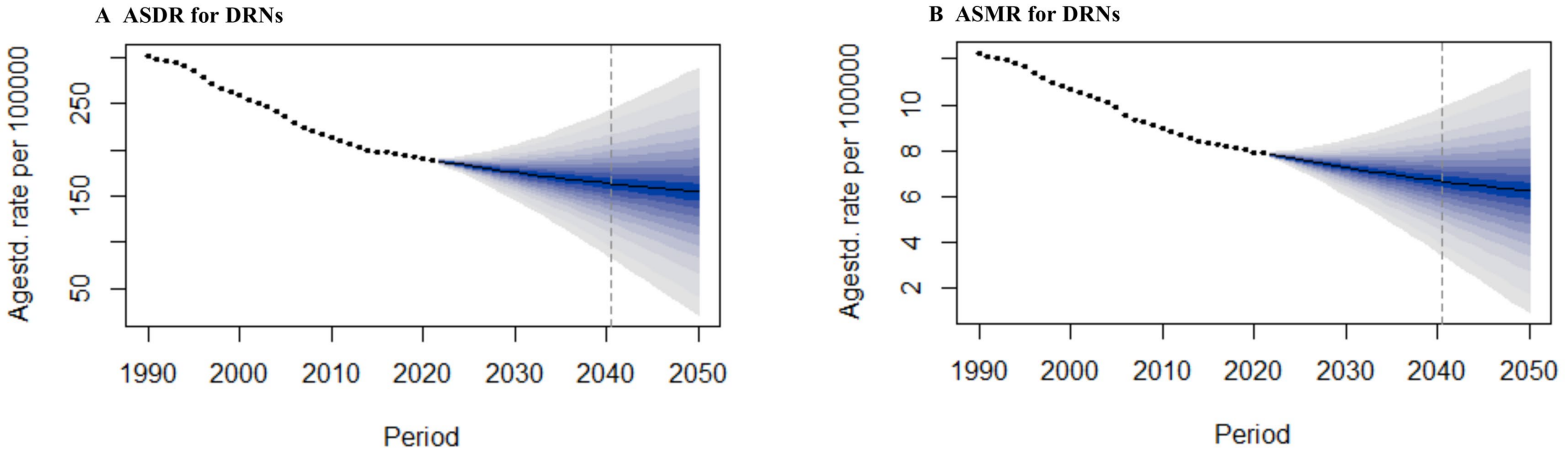
Future Forecasts of GBD in DRNs. **(A)** The ASDR for DRNs; **(B)** The ASMR for DRNs. DRNs, diet-related cancers; ASDR, age standardized DALYs rate; ASMR, age standardized mortality rate.

Figure 6 and Supplementary Figure S7 show the future forecasts of DALYs and mortality in DRNs globally. As shown in Figure 6, ASDRs and ASMRs for DRNs have declined over the past three decades and remain in a downward trend over the next 30 years. By 2050, the estimated ASDR declines to 154.57 DALYs per 100,000 population, and the ASMR is 6.24 deaths per 100,000 population (Figure 6; Supplementary Table S5). SC, CRC, and TBLC are projected to follow similar downward trends in ASDR and ASMR from 1990 to 2050 (Supplementary Figure S7; Supplementary Table S5). Especially for SC by 2050, the ASDR and ASMR decline to 12.19 DALYs per 100,000 population (a decrease of 41.34% compared with 2021) and 0.55 deaths per 100,000 population (a decrease of 37.50% compared with 2021), respectively. In contrast, for EC, BC, and PC, the ASR of DALY and mortality remain stable from 2022 to 2050 (Supplementary Figure S7; Supplementary Table S5).

### Cross-country inequality analysis

**Figure 7:**
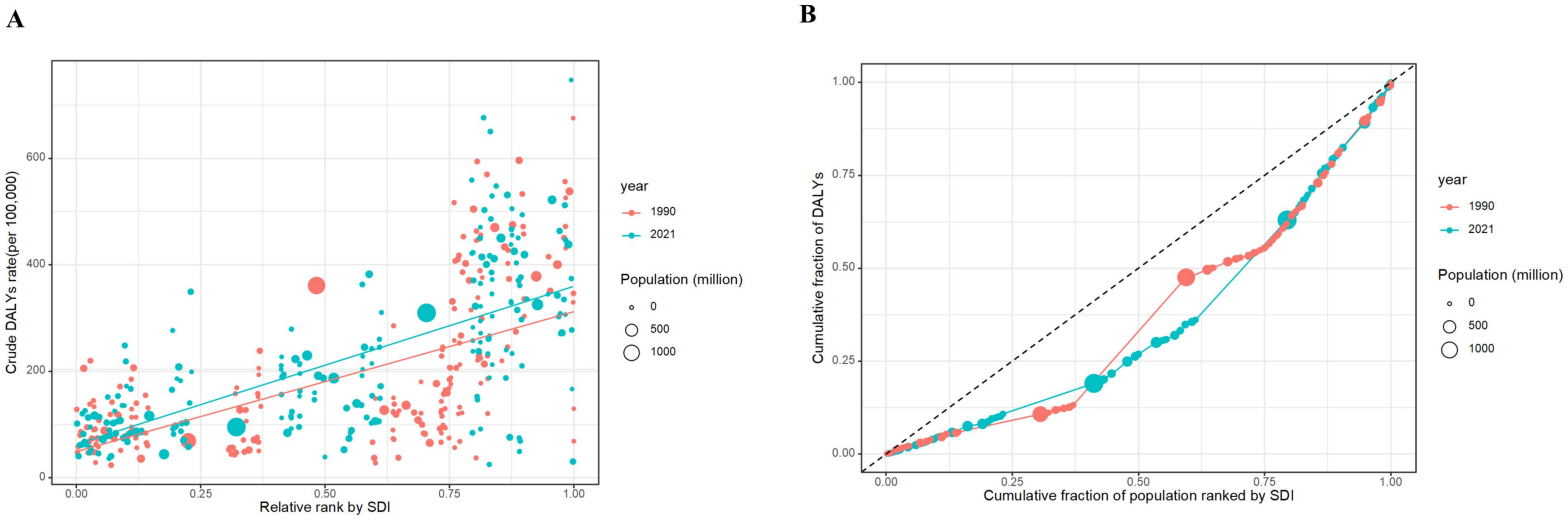
SDI-related health inequality regression and concentration curves for the DALYs of DRNs globally, 1990 and 2021. **(A)** Health inequality regression curves. **(B)** Health inequality concentration curves. DRNs, diet-related neoplasms.

In 1990 and 2021, across 204 countries and territories, remarkable absolute and relative income-related inequalities in the burden of DRNs were evident (Figure 7; Supplementary Figure S8; Supplementary Table S6). A higher DALY was disproportionately concentrated in countries with a higher SDI (Figure 7; Supplementary Table S6). As shown by the slope index of inequality, the gap in the DALY rate between the highest and lowest SDI countries was 262.74 (95% CI: 211.38 to 314.10) in 1990, and this gap further amplified to 296.14 (95% CI: 255.17 to 337.11) in 2021 (Figure 7; Supplementary Table S6). Moreover, the concentration index also showed an upward trend from 1990 to 2021, although this trend was not statistically significant (Figure 7; Supplementary Table S6). Regarding EC, the burden was comparable between high and low SDI areas. However, significant SDI-related inequalities were observed in the burden of SC, TBLC, and BC. Fortunately, these inequalities diminished by 2021. In contrast, income-related inequality in the burden of the CRC increased from 156.66 (95% CI: 125.86 to 187.46) in 1990 to 213.91 (95% CI: 186.17 to 241.65) in 2021 (Supplementary Figure S8; Supplementary Table S6).

## Discussion

Using the GBD 2021 dataset, we provide a comprehensive overview of the global burden of cancers attributable to dietary risks from 1990 to 2021. Furthermore, we project the diet-related cancer burden for the next few decades based on existing data. Our primary findings include the following: (1) the impact of dietary risk factors on common cancers has declined over the past 30 years; (2) diets high in red meat and low in whole grains and milk were major dietary risk factors for the DRNs burden; (3) the geographical heterogeneity in the burden of DRNs was considerable, and high SDI areas bear a higher DRNs burden compared to low SDI areas; and (4) the global burden of DRNs is predicted to decrease in the three decades.

According to GBD 2021, dietary risks significantly contributed to the burden of EC, SC, CRC, TBLC, and BC, while low intake of milk and calcium played a protective role in PC. Consistent with previous studies (3, 9, 22), the burden of DRNs has decreased over the past decades, a trend that may also benefit from a range of population-level dietary interventions and the increasing coverage of population-based screening projects (3, 23). These include mass media campaigns, food and menu labeling, food pricing strategies (subsidies and taxation), school procurement policies, and worksite wellness programs (3). Evidence suggests that improving diets could prevent approximately one in five deaths (3). Our findings further indicate that optimizing dietary exposures to TMREL could reduce the global cancer burden by approximately 6% (6.47% DALYs and 6.77% deaths), with CRC alone seeing a reduction of over 38% (38.74% DALYs and 38.87% deaths). This highlights the crucial role of dietary modifications in cancer prevention. As dietary interventions gain recognition as effective strategies for cancer prevention, their global implementation remains crucial (24). Targeted dietary modifications, such as reducing the consumption of red and processed meats, increasing the intake of fruits and vegetables, and promoting the adoption of Mediterranean or plant-based diets, hold significant potential to reduce the DRN burden. Furthermore, regular representative surveys on population dietary behavior and continuous DRN monitoring are essential for obtaining up-to-date country-specific data (25). Strengthening national nutrition policies, promoting public awareness campaigns, and implementing regulatory measures, such as food labeling, fiscal policies, and reformulation strategies, can further support healthier dietary patterns.

Our analysis highlighted that diets high in red meat and low in whole grains and milk are major contributors to the DRNs burden, collectively accounting for over 5% of the global cancer burden. Substantial evidence links red meat consumption to increased cancer risk, largely due to heme iron, which enhances reactive oxygen species production, particularly hydrogen peroxide, leading to inflammation, cytotoxicity, and genetic mutations that promote carcinogenesis (26, 27). Additionally, heme iron acts as a nitrosating agent, facilitating the formation of N-nitroso compounds, which induce DNA damage and adduct formation, further driving tumorigenesis (27). Conversely, whole grains have the anti-cancer properties of fiber, antioxidants, and phytochemicals (28). Cereal fiber increases fecal bulk and reduces transit time, diluting carcinogens and limiting absorption (29). Whole grain consumption, particularly wheat, promotes the production of short-chain fatty acids such as butyrate, which supports colonocyte health and induces apoptosis in cancerous cells, thereby reducing colorectal cancer risk (29, 30). Similarly, calcium, vitamin D, conjugated linoleic acid, and lactose in milk also have certain anti-tumor effects (31). Calcium may protect against colorectal cancer risk directly by increasing intra-luminal apoptosis of colonic epithelium cells as well as locally binding to secondary bile acids (32). A meta-analysis has demonstrated that increasing vegetable consumption significantly reduces esophageal cancer risk (33), with dietary fiber-derived inositol hexaphosphate suppressing tumor growth by inhibiting proliferation and promoting apoptosis (9). Low fruit consumption is a risk factor for TBLC, as fruits are rich in vitamin C and antioxidants, which may help mitigate the carcinogenic effects of tobacco exposure (34). Vitamin C reduces oxidative DNA damage and inflammation while exhibiting potential cytotoxic effects on cancer cells. Additionally, flavones in fruit regulate apoptosis by decreasing the Bcl-2/Bax ratio and suppressing NF-κB activation, thereby reducing pro-inflammatory cytokine production and tumor progression (35). For SC, a diet high in sodium is a dietary carcinogenic factor, likely due to salt-induced damage to the gastric mucosa and enhancing the carcinogenicity of nitroso compounds (36, 37). Excessive sodium consumption also promotes *Helicobacter pylori* colonization, a major risk factor for gastric cancer (37). However, diets low in milk and calcium play a protective role in PC. Consistently, previous studies have shown an increased risk of prostate cancer with intakes of milk and dietary calcium (5, 32, 38). High calcium intake may reduce bioactive vitamin D, which inhibits cancer cell proliferation and promotes apoptosis (5, 39). Similarly, milk consumption raises circulating levels of insulin-like growth factor I (IGF-1), which stimulates cell proliferation and inhibits apoptosis in both normal and cancerous prostate cells (5, 39, 40). This might explain why a diet low in milk and calcium is associated with negative ASDR and ASMR for PC.

Substantial heterogeneity in DRNs burden has been found across different world regions and nations. Central Europe had a higher ASR for diet-related cancer DALYs and deaths. Countries in this region are more likely to adopt Western dietary patterns, characterized by a higher consumption of red and processed meats, dressings, sweets, snacks, and refined grains (41, 42). Some regions in Southern Africa are converging to high-energy diets, contributing to the increasing cancer burden (43, 44). Lesotho and Zimbabwe had the highest DALYs and deaths from dietary risk, likely due to poor nutrition status and limited healthcare access (45, 46). In contrast, some countries bordering the Mediterranean Sea, such as the Syrian Arab Republic and Algeria showed a much lower burden of DRNs. The Mediterranean diet emphasizes intake of vegetables, fruits and grains, and low intake of red or processed meats and dairy products (41, 47). In a randomized controlled trial, the BC risk in the arm with a Mediterranean diet was lower than the arm with a low-fat diet (48). Although the GBD data provide valuable insights, their reliability in low SDI regions may be compromised by limited data collection capacity and potential reporting bias. In addition, most of the current experimental research on diet and cancer intervention is conducted in developed regions such as North America and Europe, while research in low SDI areas is relatively scarce (49). Despite these challenges, several studies from low-SDI regions offer empirical support for the dietary–cancer associations observed in GBD. For example, a systematic review of African studies found that consumption of red and processed meat was significantly associated with an increased risk of CRC, while high intake of fiber-rich foods such as fruits and vegetables was linked to reduced CRC risk (50). Similarly, evidence from the Middle East and North Africa region indicated that red meat consumption was associated with increased BC risk among women (51). These findings not only mirror global trends but also reinforce the biological plausibility and generalizability of GBD estimates in underrepresented regions. Expanding dietary intervention trials and improving nutritional surveillance in low-SDI settings will be crucial for enhancing data accuracy and informing region-specific cancer prevention strategies.

SDI, an indicator of socioeconomic development (41), assesses the impact of health risk factors (22). Apart from EC, high SDI locations shoulder a heavier DRNs burden than low SDI locations. In high SDI regions, the burden from red and processed meat consumption is especially significant, while in low SDI regions, it is mainly due to low vegetable intake. Less affluent groups tend to consume fewer vegetables intake than more affluent groups, likely due to higher costs and limited availability (52). Differences in food system and environment, such as policies regulating vegetable production and distribution, may also contribute to this disparity (53). A study indicated that dietary fiber plays an important role in the prevention of esophageal cancer (54). This also explains why regions with low SDI values had a higher diet-related EC burden. Since 1990, regions with higher SDIs have seen a more striking reduction in ASRs of DRNs DALYs and deaths, likely due to better education, healthcare systems, and policy priorities (41). Our cross-country inequality analysis on DRNs further proved that countries with higher SDI shouldered a disproportionately higher burden of DRNs. We also observed a significant overall increase in this inequality over time, likely driven by the global nutrition transition, whereby many low- and middle-SDI countries have shifted from traditional diets to Westernized patterns high in sodium, red and processed meat, and ultra-processed foods (55). Meanwhile, high-SDI countries have achieved larger improvements in dietary quality, health literacy, and preventive policies, whereas lower-resource settings continue to face limited access to early detection, screening programs, and timely cancer care (56, 57). Socioeconomic gradients in obesity, metabolic conditions and other mediating factors, whose prevalence has risen heterogeneously worldwide, further magnify differences in DRNs burden (58). However, the negative SII of EC further elaborates the fact that the burden of EC was concentrated in poor countries. The diminishing absolute SII values of SC, TBLC, and BC indicate a narrowing gap between high- and low-income counties. Establishing national surveillance and monitoring systems for key dietary risk factors, healthcare utilization, and significant medical expenditure plays a crucial role in this progress (59).

Sex and age differences were evident in the neoplasm burden due to dietary risk factors. Except for BC, DRNs tended to have a male predominance in terms of ASDRs and ASMRs. Men often have less healthy dietary habits, with a higher meat intake and lower fruit and vegetable consumption (22). Moreover, biological dissimilarities, including hormonal influences, also play a role in cancer development (60). Such differences should be considered in the prevention programs of national policymakers (22). When stratified by age, substantial age-related bias existed for DRNs burden. A U.S. study found higher diet-related cancer burdens in younger adults, primarily due to longer follow-up times (8). However, our findings showed a peak DRNs burden in those over 70 years. Notably, the protective effect of dietary factors on PC was more evident in older adults. Although several studies focus on the global burden of cancer attributable to risk factors, including dietary risk factors, there is a lack of age-specific data (36, 61, 62). For CRC, similar age-related differences have been reported in two previous studies that showed a higher burden in the elder population (22, 60). The reason might be longer exposure to dietary risk factors and a decrease in physiological function and immunosurveillance (16). From 1990 to 2021, although the ASRs of death and DALYs for DRNs showed a downward trend in each age group for both sexes, the absolute number of diet-related cancers has increased. Population expansion, accelerating aging, improved screening programs, and advanced diagnostic tools might account for this rise (63).

We first projected the trends of diet-related neoplasms DALYs and death through 2050. Our BAPC predictive model indicates a downward trend in global DALYs and death trends after 2022, especially in SC and CRC. However, population aging contributed to the additional dietary cancer deaths and DALYs (23). Therefore, controlling dietary risk factors and reducing the burden of DRNs requires cost-effective strategies.

### Limitations of the study

This study has some limitations. First, GBD estimates rely on multiple sources and modeling approaches, and sparse or incomplete data in many countries may introduce bias in DRN burden estimates. Second, the data on the estimated burdens of DRNs were directly obtained from the database, thus we could not verify whether there was a lag time between exposure and cancer development (13). Third, the causal link between dietary risks and disease outcomes is primarily based on primary RCTs and observational studies (15), with varying strength of evidence across foods and nutrients. Dietary data were derived from mixed sources and were unavailable for some countries, contributing to statistical uncertainty. More long-term RCTs and high-quality cohort studies are needed to strengthen the evidence base. Fourth, this analysis is based on population-level GBD data, the associations between dietary risks and cancer reported should be interpreted as ecological rather than causal, and cannot be extrapolated to individual-level risk. Finally, data on the prevalence and treatment of DRNs were not available in the GBD database.

## Conclusion

In conclusion, DRNs remain a remarkable global public health concern, although the ASDRs and ASMRs of DRNs have declined globally from 1990 to 2021. Utilizing the GBD2021 data, we conducted a comprehensive analysis of DRNs attributable to nine dietary risks, with diets high in red meat and low in whole grains and milk identified as primary dietary risk factors. Notably, a diet low in milk and calcium may play a protective role for PC. The changing pattern of DRNs burden was heterogeneous in relation to sex, region, and age, with those over 70 years old needing to pay more attention to their dietary structure. Our projections suggest that the global ASR of DRNs might tend to decline until 2050. Moreover, DRNs exhibited significant income-related inequality, disproportionately burdening more affluent locations. Therefore, optimizing dietary intake could be particularly important for reducing disparities related to DRNs, especially in CRC, which has the largest number of diet-attributable cases. The findings provide valuable insights for policymakers to tailor dietary guidelines based on the specific circumstances of different countries. Building on these findings, future research should focus on evaluating the effectiveness of targeted dietary interventions and incorporating additional dietary and lifestyle risk factors, such as overall dietary patterns, obesity, physical inactivity, metabolic disorders, and potential interactions with the gut microbiome, to better understand region-specific determinants of DRNs and guide the development of effective prevention strategies.

## Data Availability

Publicly available datasets were analyzed in this study. This data can be found at: http://ghdx.healthdata.org/gbd-2021.

## Footnotes

1
https://ghdx.healthdata.org/record/ihme-data/global-population-forecasts-2017-2100
