# Supplementary material for: Global burden and trends analysis of common cancers attributable to dietary risks from 1990 to 2021 and projection to 2050: a secondary analysis for the global burden of disease study 2021
Source: Front Nutr. 2025 Dec 19;12:1673422. doi: 10.3389/fnut.2025.1673422 (PMC12757258; doi:10.3389/fnut.2025.1673422)
Supplement: Supplementary file 1 [file Table_1.DOCX]

**Figure S1**. Rank changes for dietary risk -attributable to **(A)** all causes or **(B)** neoplasms and percentage change in age-standardized DALY rates globally from 1990 to 2021.


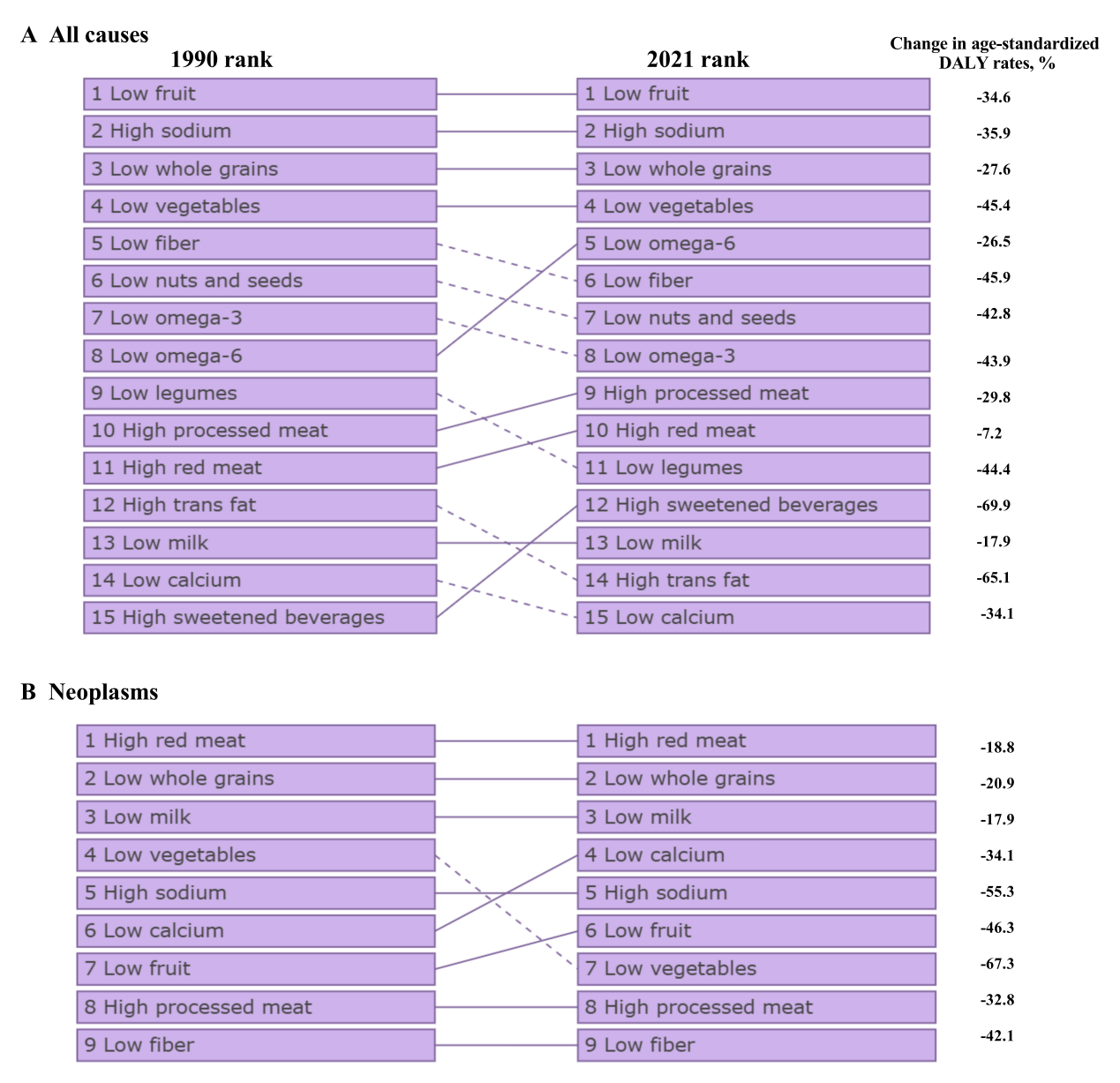


**Figure S2** Diet-related ASR from neoplasms due to disease groups (left) and risk factors (right) in 2021. **(A)** The ASDR per 100,000 in 2021, both sexes. **(B)** The ASMR per 100,000 in 2021, both sexes. ASDR: age standardized DALYs rate; ASMR: age standardized mortality rate.


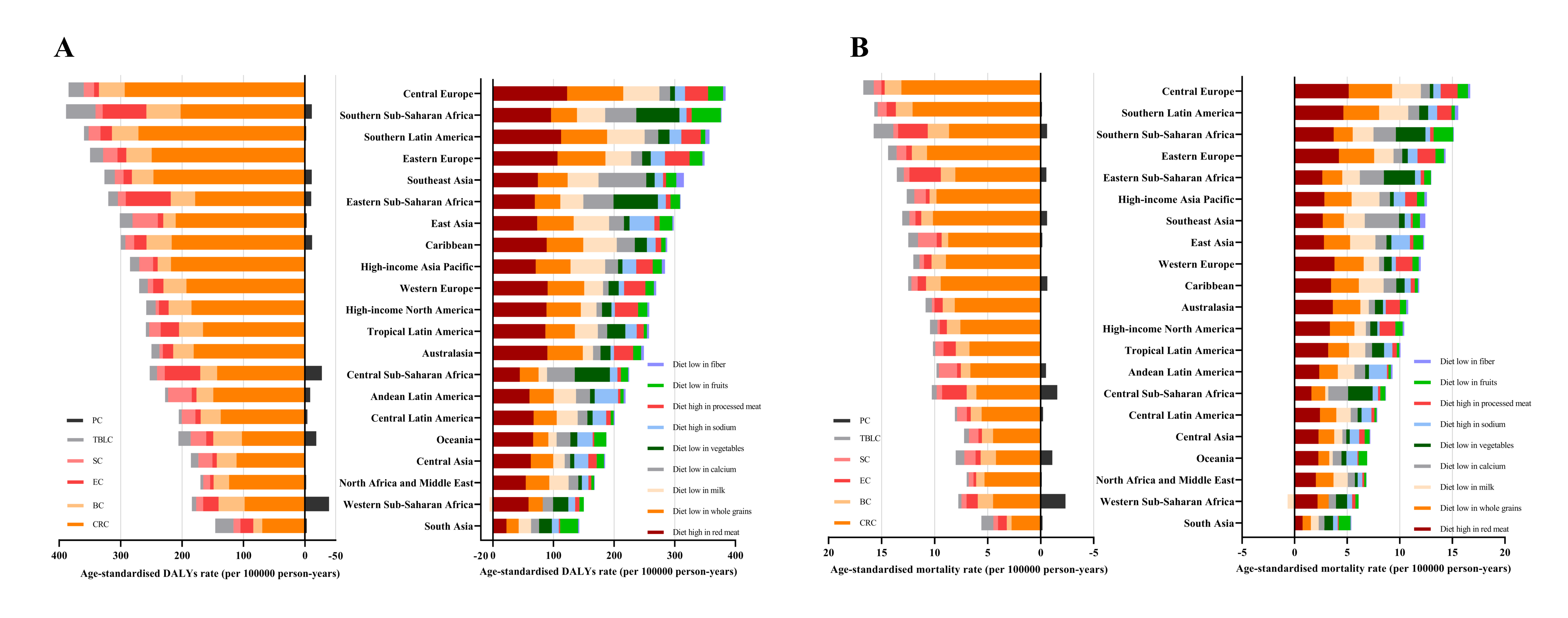


**Figure S3**. The estimated annual percentage change (EAPC) for **(A)** ASDR and **(B)** ASMR of in 21 global burden of disease regions classified by 5 sociodemographic index levels for both sexes in 1990-2021. ASDR: age-standardized DALYs rates; ASMR: age-standardized mortality rates.


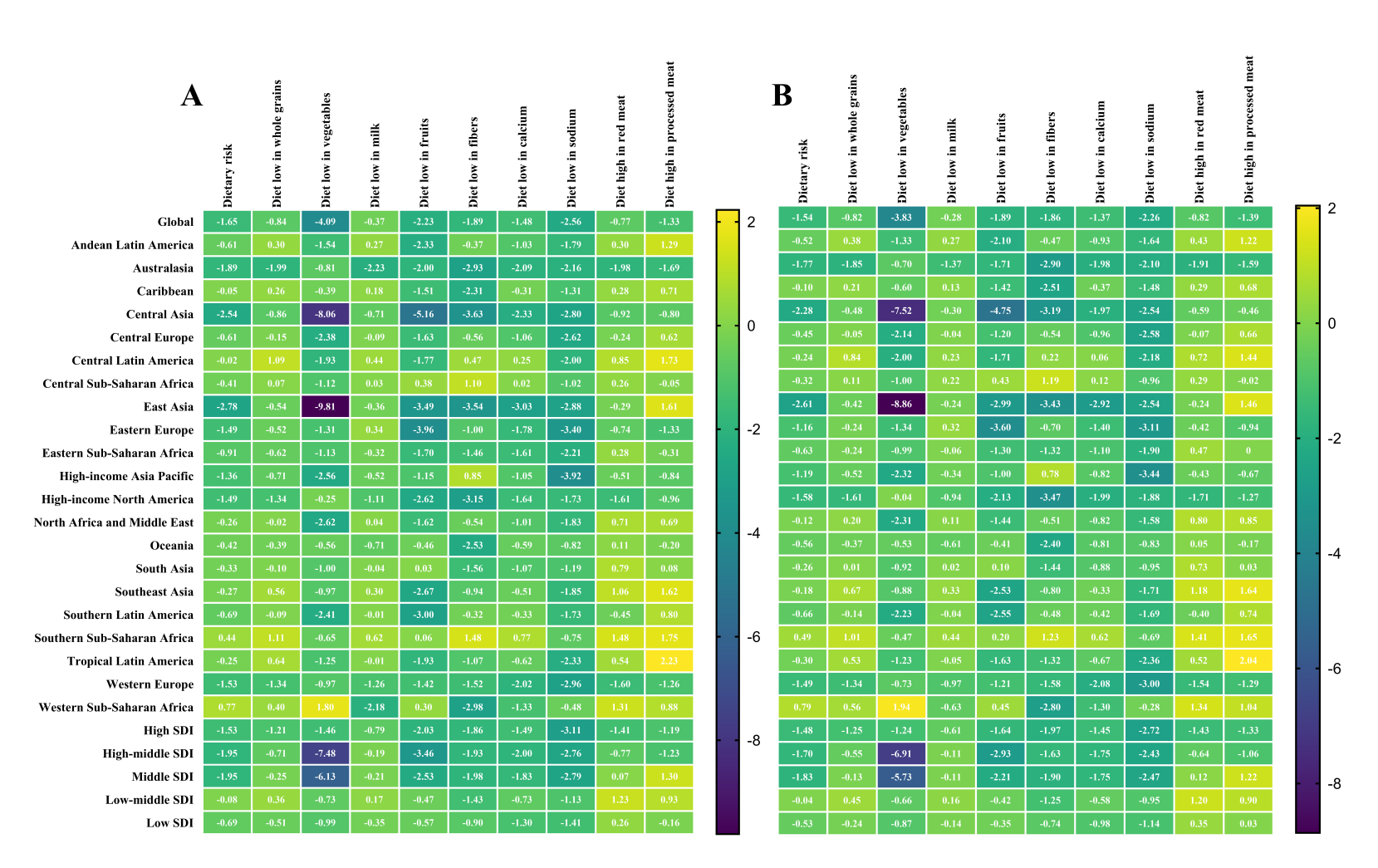


**Figure S4**. SDI difference in ASR for dietary risks attributed to the global burden of neoplasms. **(A)** ASDR and ASMR dependent on SDI for dietary risks attributed to the global burden of neoplasms in both sexes. **(B)** SDI-stratified annual percentage change of ASDR and ASMR for dietary risks attributed to the global burden of neoplasms from 1990 to 2021. ASDR: age-standardized DALYs rates; ASMR: age-standardized mortality rates; SDI: sociodemographic index.


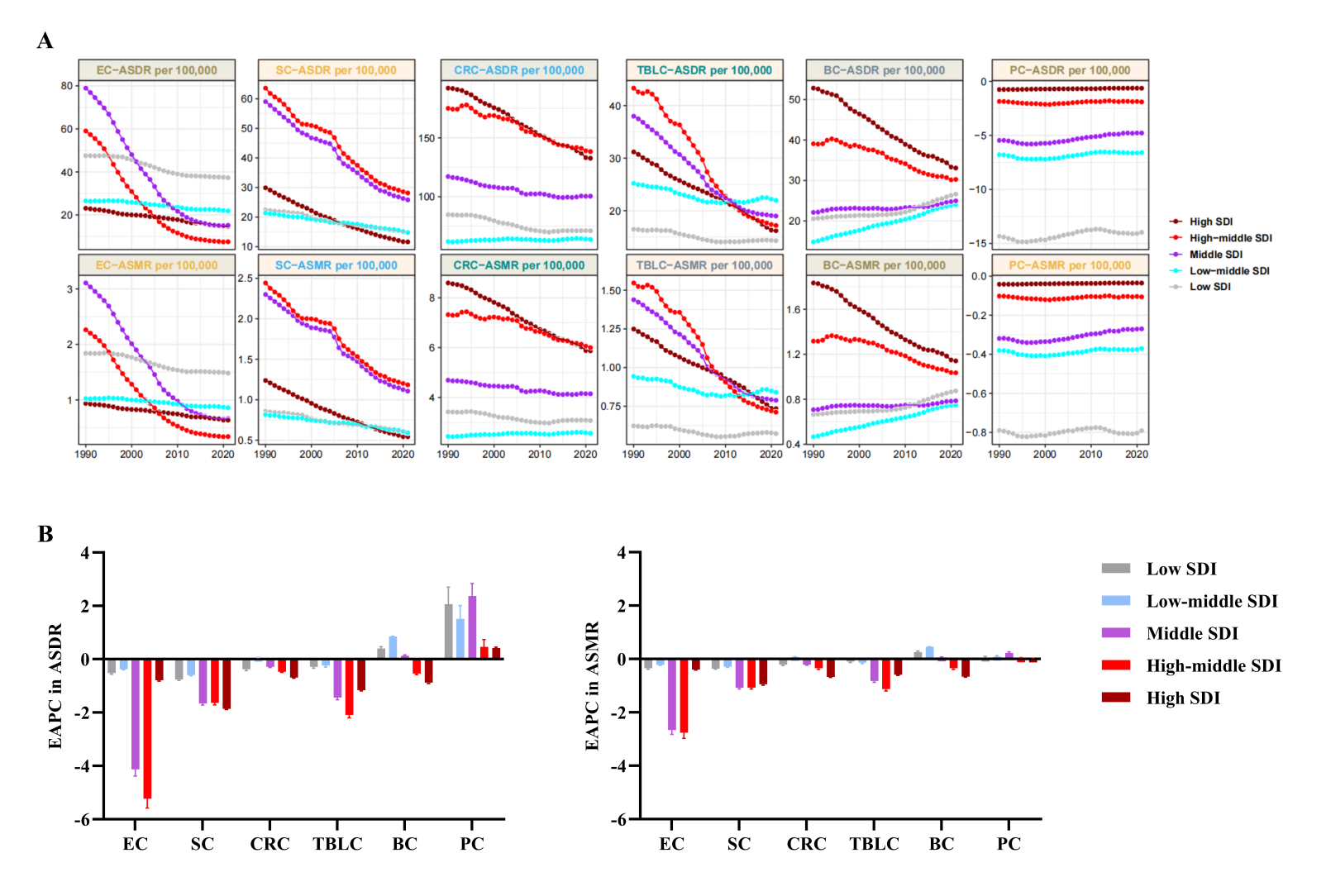


**Figure S5**. Sex difference in ASR for dietary risks attributed to the global burden of neoplasms. **(A)** Sex difference in ASDR and ASMR for dietary risks attributed to the global burden of neoplasms. **(B)** Sex-stratified annual percentage change of ASDR and ASMR for dietary risks attributed to the global burden of neoplasms from 1990 to 2021. ASDR: age-standardized DALYs rates; ASMR: age-standardized mortality rates.


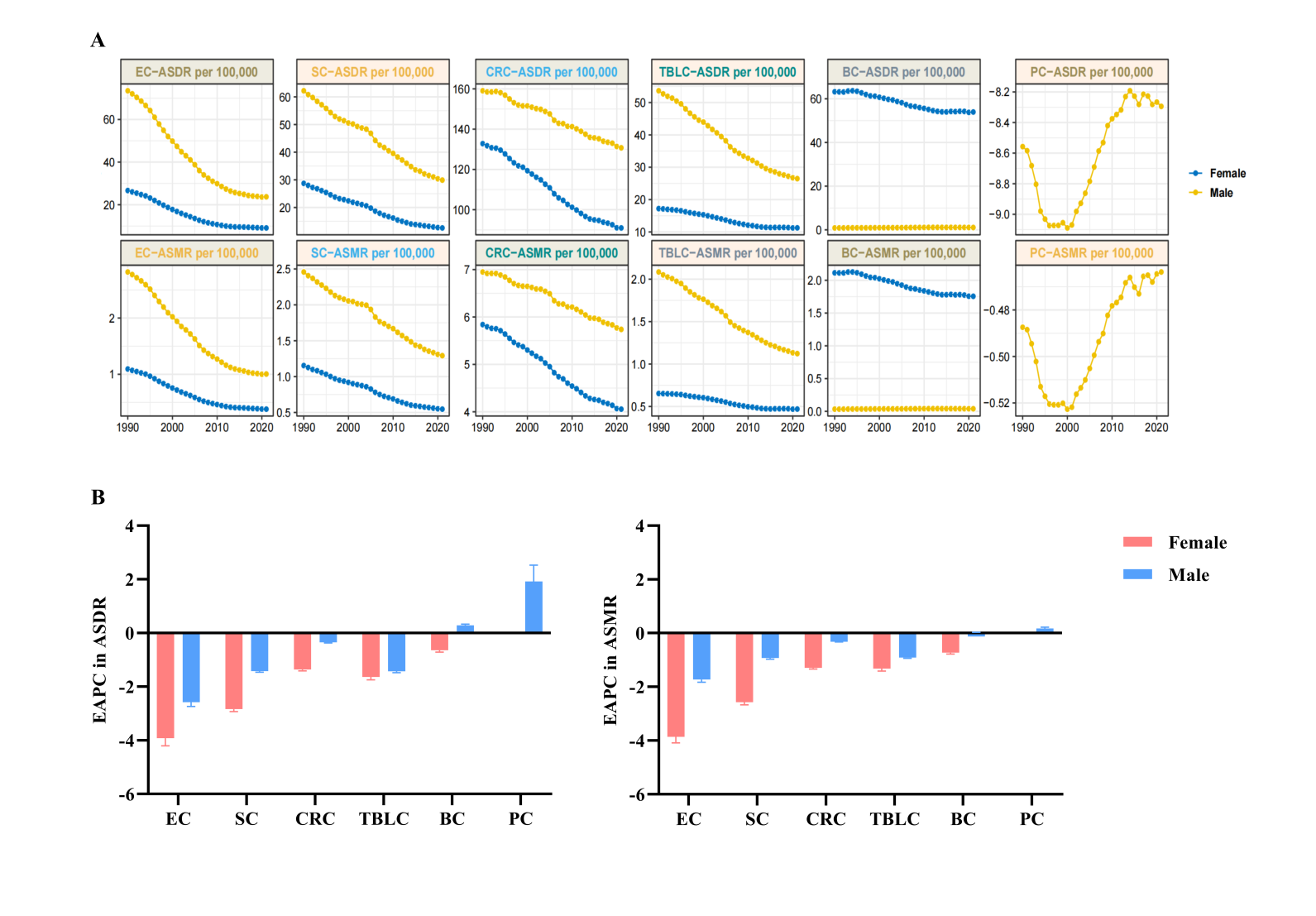


**Figure S6**. Age difference in ASR for dietary risks attributed to the global burden of neoplasms. **(A)** Age difference in ASDR and ASMR for dietary risks attributed to the global burden of neoplasms in both sexes. **(B)** Age-stratified annual percentage change of ASDR and ASMR for dietary risks attributed to the global burden of neoplasms in both sexes from 1990 to 2021. ASDR: age-standardized DALYs rates; ASMR: age-standardized mortality rates.


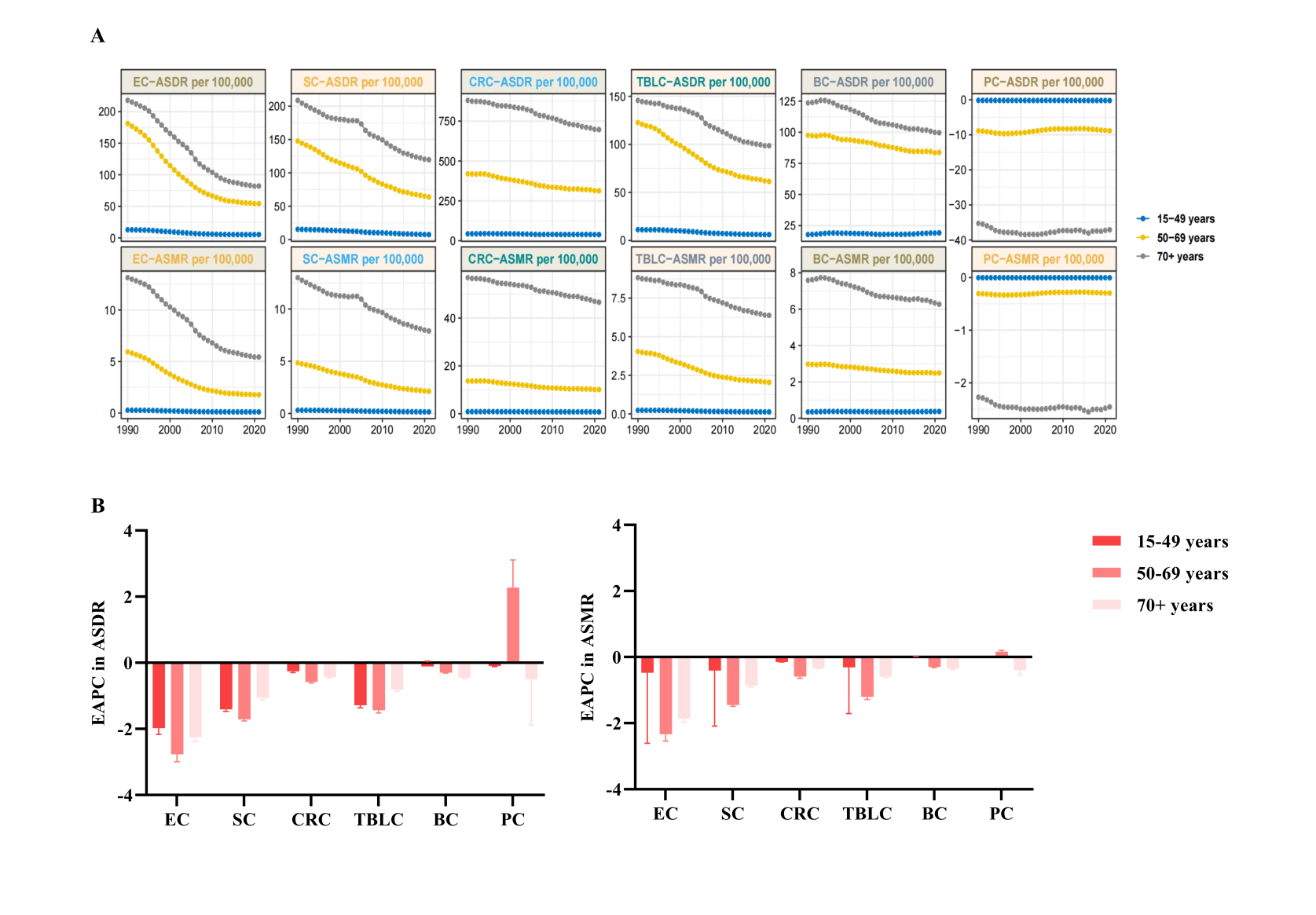


**Figure S7**. Temporal trends of for ASMR and ASDR for EC**(A-B)**, SC **(C–D)**, CRC **(E-F)**, TBLC **(G-H)**, BC **(I-J)** and PC **(K-L)** worldwide, 1990 and 2021. ASDR: age standardized DALYs rate; ASMR: age standardized mortality rate; DRNs: diet-related cancers; EC: esophageal cancer; SC: stomach cancer; CRC: colon and rectum cancer; TBLC: tracheal, bronchus, and lung cancer; BC: breast cancer; PC: prostate cancer.


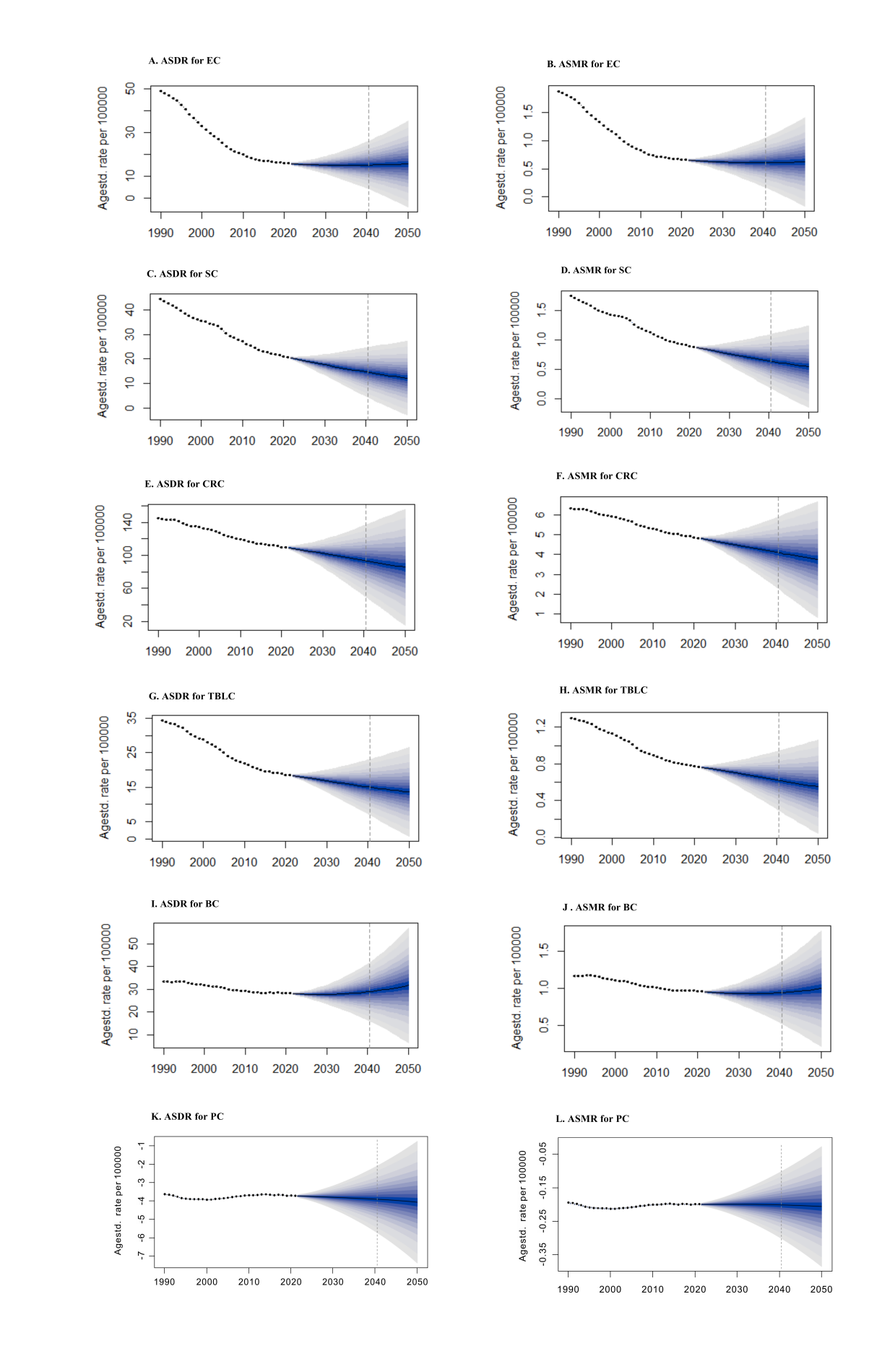


**Figure S8**. Health inequality regression curves and concentration curves for the DALYs of EC**(A-B)**, SC **(C–D)**, CRC **(E-F)**, TBLC **(G-H)**, BC **(I-J)** and PC **(K-L)** worldwide, 1990 and 2021. DALYs: disability-adjusted life-years; EC: esophageal cancer; SC: stomach cancer; CRC: colon and rectum cancer; TBLC: tracheal, bronchus, and lung cancer; BC: breast cancer; PC: Prostate cancer.


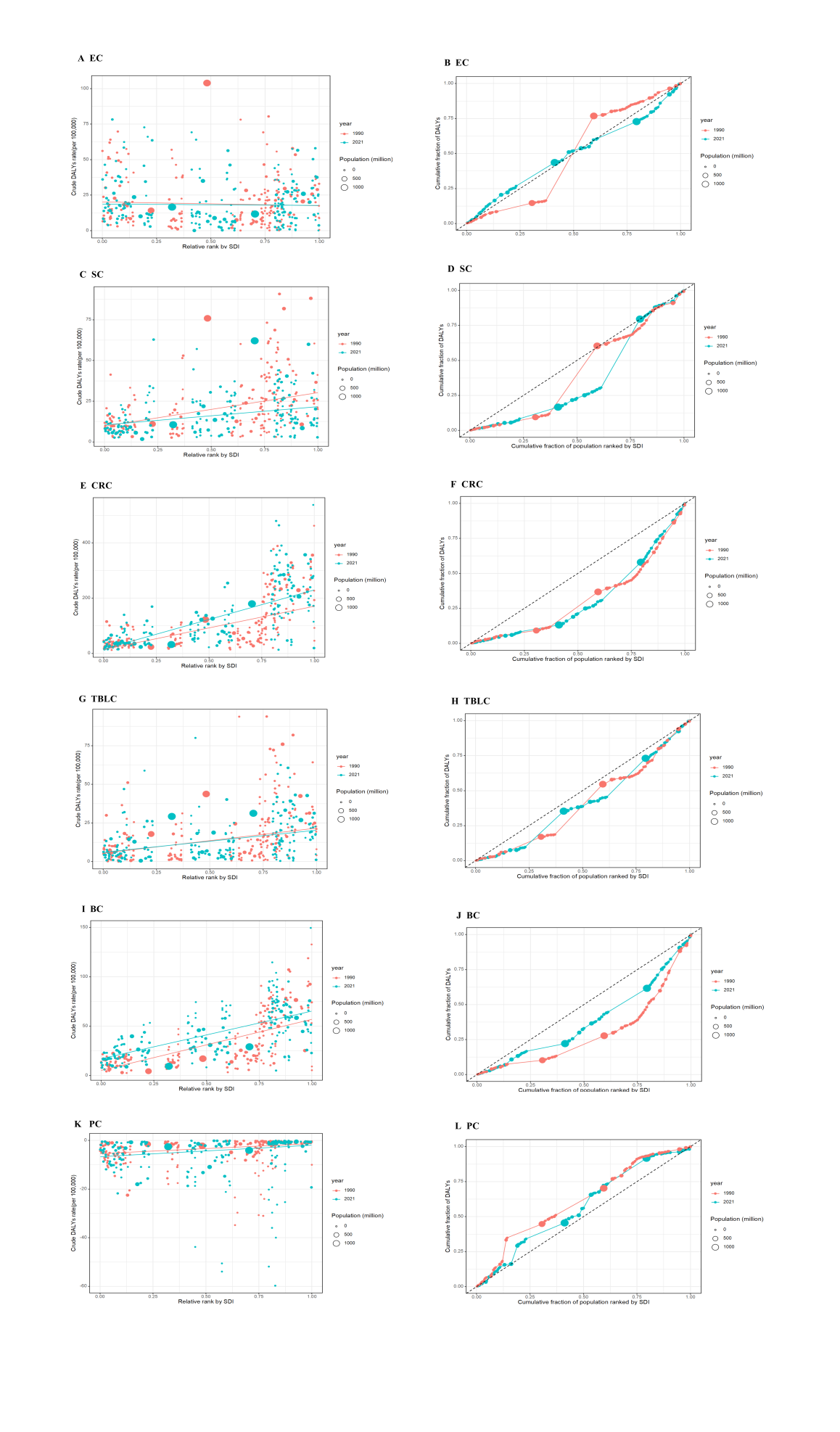


**Table S1**: The definition and theoretical minimum risk exposure level for dietary risk factor related to neoplasms in 2021. (Source: GBD 2021 Risk factors study)

| **Risk factors** | **Definition** | **Theoretical minimum risk exposure level** |
| --- | --- | --- |
| **Diet low in whole grains** | Average daily consumption of whole grains (bran, germ, and endosperm in their natural proportion) from breakfast cereals, bread, rice, pasta, biscuits, muffins, tortillas, pancakes, and other sources. | Consumption of whole grains 160-210 g /day |
| **Diet low in milk** | Average daily consumption of milk, including non-fat, low-fat, and full-fat milk, excluding soy milk and other plant derivatives. | Consumption of milk 280–340 g/day (males)；500–610 g/day (females) |
| **Diet low in calcium** | Average daily consumption of calcium from all sources, including milk, yogurt, and cheese. | Consumption of calcium 0.72–0.86 g/day (males)；1.06–1.2 g/day (females) |
| **Diet low in fruit** | Average daily consumption of fruit including fresh, frozen, cooked, canned, or dried fruit, excluding fruit juices and salted or pickled fruits. | Consumption of fruit 340–350 g/day |
| **Diet high in red meat** | Average daily consumption of red meat (beef, pork, lamb, and goat but excluding poultry, fish, eggs, and all processed meats). | Consumption of red meat 0–200 g/day |
| **Diet high in sodium** | Average daily consumption of sodium. | Consumption of sodium of 1-5 g/day |
| **Diet high in processed meat** | Average daily consumption of meat preserved by smoking, curing, salting, or addition of chemical preservatives. | Consumption of processed meat 0 g/day |
| **Diet low in vegetables** | Average daily consumption of vegetables, including fresh, frozen, cooked, canned, or dried vegetables and excluding legumes and salted or pickled vegetables, juices, nuts and seeds, and starchy vegetables such as potatoes or corn. | Consumption of vegetables 306–372 g/day |
| **Diet low in fiber** | Average daily consumption of fiber from all sources including fruits, vegetables, grains, legumes, and pulses. | Consumption of fibre 22–25 g/day |

**Table S2: Diet-related DALYs and deaths globally in 2021 due to (a) disease group and (b) dietary risks.**

|  | **DALYs** | | |  | **Deaths** | | |  | **DALYs** | | |  | **Deaths** | | | | |
| --- | --- | --- | --- | --- | --- | --- | --- | --- | --- | --- | --- | --- | --- | --- | --- | --- | --- |
|  | **Number (95% UI)** | **ASR (95% UI)** | **PAF (%)** |  | **Number (95% UI)** | **ASR (95% UI)** | **PAF (%)** |  | **Number (95% UI)** | **ASR (95% UI)** | **PAF (%)** |  | **Number (95% UI)** | | **ASR (95% UI)** | **PAF (%)** | |
| **(a) Due to disease group** |  |  |  |  |  |  |  |  |  |  |  |  |  | |  |  | |
| All causes | 178,258,703 (49,769,061-260,914,616) | 2,074.81 (579.23-3,038.38) | 6.19 (1.76-9.14) |  | 7,219,016 (1,961,200-10,767,030) | 86.26 (23.35-128.98) | 10.63 (2.90-15.94) |  | - | - |  |  | - | | - |  | |
| Cardiovascular diseases | 134,179,728 (32,581,870-192,859,591) | 1,563.86 (378.95-2,246.75) | 31.31 (7.80-45.21) |  | 5,833,851 (1,357,129-8,661,541) | 69.81 (16.19-104.09) | 30.02 (7.31-44.59) |  | - | - |  |  | - | | - |  | |
| Diabetes and kidney diseases | 27,118,091 (10,638,211-41,521,373) | 314.86 (124.01-481.70) | 21.88 (8.62-32.75) |  | 698,425 (336,903-1,008,081) | 8.35 (4.03-12.05) | 21.85  (10.71-31.46) |  | - | - |  |  | - | | - |  | |
| Neoplasms | 16,403,609 (4,939,614-29,035,004) | 189.62 (57.13-335.37) | 6.47 (2.01-11.53) |  | 669,656 (207,111-1,176,737) | 7.90 (2.45-13.85) | 6.77 (2.20-12.05) |  | - | - |  |  | - | | - |  | |
| Colon and rectum cancer | - | - | - |  | - | - | - |  | 9,458,464 (3,251,668-14,521,174) | 109.71 (37.68-168.52) | 38.74 (13.49-58.50) |  | 406,099 (138,065-628,056) | | 4.82 (1.64-7.46) | 38.87 (13.38-58.80) | |
| Breast cancer | - | - | - |  | - | - | - |  | 2,451,719 (-791-5,232,217) | 28.37 (-0.01-60.54) | 11.86 (-0.00-25.33) |  | 81,506 (-26-175,445) | | 0.96 (0.00-2.06) | 12.07 (-0.00-25.79) | |
| Esophageal cancer | - | - | - |  | - | - | - |  | 1,396,852 (-293,021-2,888,420) | 16.02 (-3.37-33.09) | 10.74 (-2.37-21.87) |  | 56,939 (-12,023-118,370) | | 0.66 (-0.14-1.38) | 10.56 (-2.35-21.62) | |
| Stomach cancer | - | - | - |  | - | - | - |  | 1,804,592 (0-8,884,379) | 20.78 (0.00-102.38) | 7.93 (-0.00-40.17) |  | 75,661 (0-372,194) | | 0.89 (0.00-4.37) | 7.94 (-0.00-40.24) | |
| Tracheal, bronchus, and lung cancer | - | - | - |  | - | - | - |  | 1,611,267 (828,054-2,347,369) | 18.46 (9.49-26.90) | 3.46 (1.83-4.93) |  | 66,045 (34,006-97,033) | | 0.77 (0.40-1.13) | 3.27 (1.72-4.70) | |
| Prostate cancer | - | - | - |  | - | - | - |  | -319,285 (-854,392-193,101) | -3.73 (-9.97-2.25) | -3.91 (-10.26-2.33) |  | -16,595 (-44,502-9,928) | | -0.20 (-0.53-0.12) | -3.83 (-10.09-2.28) | |
| Other causes | 557,276 (120,597-999,285) | 6.46 (1.40-11.58) | 0.07 (0.01-0.12) |  | 17,083 (3,642-30,908) | 0.20 (0.04-0.36) | 0.10 (0.02-0.18) |  | - | - |  | - | - | | - |  | |
| **(b) Due to dietary risks** |  |  |  |  |  |  |  |  |  |  |  |  |  | |  |  | |
| Diet high in processed meat | 10,357,091 (2,261,202-17,731,836) | 120.21 (26.25-205.71) | 0.36 (0.08-0.61) |  | 295,269 (56,805-502,317) | 3.53 (0.68-6.01) | 0.43 (0.08-0.74) |  | 1,301,644 (-310,251-2,664,056) | 15.11 (-3.60-30.93) | 0.51 (-0.12-1.05) |  | 57,149 (-13,412-117,695) | | 0.68 (-0.16-1.40) | 0.58 (-0.14-1.18) | |
| Diet high in red meat | 9,633,539 (-4,909-13,030,228) | 111.94 (-0.06-151.09) | 0.33 (-0.00-0.44) |  | 333,872 (-146-444,431) | 4.01 (0.00-5.37) | 0.49 (-0.00-0.66) |  | 6,003,957 (-1,387-12,291,168) | 69.56 (-0.02-142.38) | 2.37 (-0.00-4.81) |  | 234,492 (-53-481,528) | | 2.77 (0.00-5.70) | 2.37 (-0.00-4.82) | |
| Diet high in sodium | 41,275,914 (9,297,839-91,456,678) | 478.29 (105.88-1,064.85) | 1.44 (0.32-3.23) |  | 1,857,696 (367,761-4,251,577) | 22.12 (4.25-50.87) | 2.74 (0.54-6.21) |  | 1,804,592 (0-8,884,379) | 20.78 (0.00-102.38) | 0.71 (-0.00-3.55) |  | 75,661 (0-372,194) | | 0.89 (0.00-4.37) | 0.76 (-0.00-3.82) | |
| Diet low in calcium | 2,031,478 (1,558,143-2,498,108) | 23.56 (18.11-28.95) | 0.07 (0.05-0.09) |  | 83,864 (64,826-102,766) | 0.99 (0.76-1.22) | 0.12 (0.10-0.15) |  | 2,031,478 (1,558,143-2,498,108) | 23.56 (18.11-28.95) | 0.80 (0.63-0.97) |  | 83,864 (64,826-102,766) | | 0.99 (0.76-1.22) | 0.85 (0.68-1.02) | |
| Diet low in fiber | 16,696,607 (5,749,429-26,277,842) | 195.12 (67.53-306.51) | 0.58 (0.20-0.92) |  | 618,783 (226,107-987,569) | 7.38 (2.74-11.80) | 0.91 (0.34-1.44) |  | 305,676 (135,089-469,863) | 3.58 (1.58-5.50) | 0.12 (0.06-0.18) |  | 13,145 (5,762-20,265) | | 0.16 (0.07-0.24) | 0.13 (0.06-0.20) | |
| Diet low in fruits | 43,766,703 (17,669,818-65,157,800) | 510.10 (207.69-758.19) | 1.52 (0.61-2.27) |  | 1,683,592 (769,758-2,470,250) | 20.07 (9.34-29.32) | 2.48 (1.14-3.62) |  | 1,611,267 (828,054-2,347,369) | 18.46 (9.49-26.90) | 0.64 (0.34-0.90) |  | 66,045 (34,006-97,033) | | 0.77 (0.40-1.13) | 0.67 (0.35-0.95) | |
| Diet low in milk | 3,492,706 (1,198,015-5,497,563) | 40.50 (13.91-63.72) | 0.12 (0.04-0.19) |  | 146,611 (52,324-230,010) | 1.74 (0.62-2.73) | 0.22 (0.08-0.33) |  | 3,492,706 (1,198,015-5,497,563) | 40.50 (13.91-63.72) | 1.38 (0.47-2.14) |  | 146,611 (52,324-230,010) | | 1.74 (0.62-2.73) | 1.48 (0.53-2.27) | |
| Diet low in vegetables | 20,655,691 (12,448,914-28,673,802) | 241.70 (146.31-335.38) | 0.72 (0.44-1.00) |  | 861,480 (541,278-1,189,503) | 10.35 (6.56-14.30) | 1.27 (0.79-1.74) |  | 1,396,852 (-293,021-2,888,420) | 16.02 (-3.37-33.09) | 0.55 (-0.12-1.11) |  | 56,939 (-12,023-118,370) | | 0.66 (-0.14-1.38) | 0.57 (-0.13-1.17) | |
| Diet low in whole grains | 40,414,150 (16,951,268-60,948,337) | 470.13 (198.27-709.93) | 1.40 (0.60-2.13) |  | 1,546,243 (707,297-2,343,822) | 18.43 (8.47-27.95) | 2.28 (1.05-3.42) |  | 4,327,219 (1,754,865-6,578,232) | 50.19 (20.37-76.30) | 1.71 (0.71-2.50) |  | 186,257 (76,127-284,803) | | 2.21 (0.91-3.38) | 1.88 (0.79-2.77) | |
| Diet high in sugar-sweetened beverages | 3,607,897 (1,703,481-5,566,160) | 41.84 (19.73-64.58) | 0.12 (0.06-0.19) |  | 75,725 (34,284-117,266) | 0.90 (0.41-1.40) | 0.11 (0.05-0.17) |  | - | - | - |  | - | | - | - | |
| Diet high in trans fatty acids | 2,491,995 (241,562-4,781,961) | 28.92 (2.81-55.44) | 0.09 (0.01-0.17) |  | 93,311 (9,229-179,614) | 1.10 (0.11-2.12) | 0.14 (0.01-0.26) |  | - | - | - |  | - | | - | - | |
| Diet low in legumes | 9,162,567 (-7,635,025-22,951,528) | 107.02 (-89.10-268.59) | 0.32 (-0.26-0.80) |  | 389,329 (-313,415-1,011,098) | 4.67 (-3.75-12.16) | 0.57 (-0.46-1.49) |  | - | - | - |  | - | | - | - | |
| Diet low in nuts and seeds | 16,261,043 (4,808,156-26,189,582) | 189.88 (56.11-305.65) | 0.56 (0.17-0.92) |  | 658,247 (184,906-1,086,134) | 7.86 (2.20-12.99) | 0.97 (0.27-1.60) |  | - | - | - |  | - | | - | - | |
| Diet low in polyunsaturated fatty acids | 17,869,861 (-56,003,352-65,962,499) | 208.15 (-651.94-768.84) | 0.62 (-1.99-2.29) |  | 737,879 (-2,118,790-2,767,688) | 8.80 (-25.14-33.08) | 1.08 (-3.15-4.10) |  | - | - | - |  | - | | - | - | |
| Diet low in seafood omega-3 fatty acids | 15,511,021 (3,098,824-25,946,111) | 181.07 (36.18-302.84) | 0.54 (0.11-0.91) |  | 627,342 (119,538-1,082,745) | 7.49 (1.42-12.95) | 0.92 (0.17-1.60) |  | - | - | - |  | - | | - | - | |
| Abbreviations: PAF, population attributable fraction; ASR, age standardized rate; UI, uncertainty interval. | | | | | | | | | | | | | |  | | |  |

**Table S3**. DALYs and deaths attributable to diet-related neoplasms in 21 regions in 2021.

| **Regions** | **DALYs** | |  | **Deaths** | |
| --- | --- | --- | --- | --- | --- |
|  | **Number (95% UI)** | **ASR (95% UI)** |  | **Number (95% UI)** | **ASR (95% UI)** |
| Andean Latin America | 95,068 (28,062-220,263) | 156.76 (47.39-363.24) |  | 3,808 (1,250-8,808) | 6.53 (2.18-15.09) |
| Australasia | 99,976 (21,293-169,888) | 199.41 (41.75-342.84) |  | 4,772 (1,055-7,960) | 8.56 (1.89-14.34) |
| Caribbean | 106,132 (35,183-180,166) | 198.15 (65.72-336.61) |  | 4,290 (1,561-7,171) | 7.94 (2.89-13.28) |
| Central Asia | 131,983 (31,655-262,828) | 149.44 (36.27-296.26) |  | 4,604 (1,140-9,092) | 5.76 (1.44-11.34) |
| Central Europe | 597,272 (157,647-1,004,078) | 282.90 (73.39-477.40) |  | 27,497 (7,528-45,889) | 12.13 (3.28-20.29) |
| Central Latin America | 380,935 (103,927-756,723) | 147.76 (40.6-294.02) |  | 14,150 (4,069-28,517) | 5.70 (1.66-11.50) |
| Central Sub-Saharan Africa | 111,902 (33,200-191,396) | 167.75 (61.53-276.15) |  | 3,515 (1,263-5,800) | 6.26 (2.81-10.06) |
| East Asia | 4,609,040 (1,540,767-9,434,185) | 212.63 (71.3-431.73) |  | 184,512 (63,447-379,018) | 8.74 (3.01-17.81) |
| Eastern Europe | 913,592 (232,096-1,615,698) | 267.79 (67.71-474.80) |  | 38,364 (10,072-67,071) | 10.84 (2.84-19.01) |
| Eastern Sub-Saharan Africa | 457,927 (106,901-754,926) | 239.89 (61.55-387.71) |  | 15,618 (4,089-25,132) | 9.88 (2.93-15.69) |
| High-income Asia Pacific | 820,116 (243,677-1,506,385) | 194.47 (56.39-356.77) |  | 44,667 (13,952-81,519) | 8.53 (2.55-15.72) |
| High-income North America | 1,210,701 (229,601-2,069,819) | 200.96 (37.31-345.12) |  | 53,160 (10,817-89,622) | 8.06 (1.61-13.6) |
| North Africa and Middle East | 608,559 (178,260-1,102,172) | 120.95 (36.64-218.53) |  | 21,079 (6,544-38,075) | 4.84 (1.57-8.62) |
| Oceania | 13,587 (4,218-28,063) | 145.69 (51.89-302.24) |  | 407 (146-844) | 5.19 (1.92-10.77) |
| South Asia | 1,792,221 (570,876-3,098,564) | 112.31 (36.66-193.32) |  | 62,053 (21,041-106,201) | 4.21 (1.48-7.20) |
| Southeast Asia | 1,497,633 (683,700-2,400,293) | 211.69 (100.22-334.64) |  | 52,421 (25,993-81,636) | 8.16 (4.23-12.43) |
| Southern Latin America | 214,725 (55,366-370,705) | 251.76 (64.38-434.85) |  | 9,598 (2,582-16,523) | 10.84 (2.91-18.68) |
| Southern Sub-Saharan Africa | 187,817 (51,351-312,283) | 297.88 (83.75-492.82) |  | 6,561 (1,905-10,755) | 11.74 (3.52-19.17) |
| Tropical Latin America | 516,705 (109,872-934,440) | 197.23 (42.15-356.50) |  | 19,535 (4,330-35,104) | 7.64 (1.7-13.73) |
| Western Europe | 1,782,082 (348,757-3,040,044) | 206.89 (39.20-357.56) |  | 91,312 (18,988-155,345) | 9.07 (1.84-15.5) |
| Western Sub-Saharan Africa | 255,636 (80,899-448,443) | 103.80 (34.10-183.58) |  | 7,734 (2,505-13,620) | 3.53 (0.69-6.27) |
| Abbreviations: ASR, age standardized rate; UI, uncertainty interval. | | | | | |

**Table S4**. DALYs and deaths attributable to diet-related neoplasms in 204 countries and territories in 2021.

| **Regions** | **DALYs** | |  | **Deaths** | |
| --- | --- | --- | --- | --- | --- |
|  | **Number (95% UI)** | **ASR (95% UI)** |  | **Number (95% UI)** | **ASR (95% UI)** |
| Afghanistan | 35,382 (7,347-77,665) | 283.20 (61.73-609.37) |  | 1,065 (237-2,296) | 10.55 (2.51-22.12) |
| Albania | 4,590 (926-9,891) | 109.90 (21.58-236.40) |  | 211 (45-453) | 4.92 (1.02-10.55) |
| Algeria | 23,360 (5,224-44,497) | 61.97 (14.54-115.78) |  | 850 (211-1,566) | 2.67 (0.70-4.85) |
| American Samoa | 128 (42-242) | 253.27 (87.63-482.03) |  | 4 (2-8) | 9.62 (3.45-18.14) |
| Andorra | 295 (62-530) | 193.84 (40.35-349.33) |  | 13 (3-24) | 8.42 (1.82-14.99) |
| Angola | 27,161 (6,643-49,959) | 189.51 (54.65-336.31) |  | 850 (239-1,517) | 7.26 (2.46-12.20) |
| Antigua and Barbuda | 228 (57-413) | 208.71 (53.16-376.91) |  | 9 (2-16) | 8.98 (2.49-16.12) |
| Argentina | 146,620 (34,811-255,045) | 267.84 (62.98-466.91) |  | 6,443 (1,634-11,081) | 11.37 (2.86-19.58) |
| Armenia | 7,792 (1,888-14,627) | 181.94 (43.70-341.21) |  | 330 (85-616) | 7.61 (1.95-14.17) |
| Australia | 81,307 (16,452-138,154) | 193.08 (38.43-331.08) |  | 3,884 (813-6,516) | 8.25 (1.73-13.91) |
| Austria | 26,689 (5,168-46,908) | 156.52 (29.36-276.37) |  | 1,349 (277-2,366) | 6.94 (1.40-12.19) |
| Azerbaijan | 16,179 (3,434-35,550) | 142.85 (30.37-314.92) |  | 550 (118-1,226) | 5.44 (1.16-12.18) |
| Bahamas | 1,177 (358-2,141) | 270.98 (88.04-488.92) |  | 41 (15-72) | 10.11 (3.89-17.62) |
| Bahrain | 1,404 (292-2,532) | 132.17 (31.19-234.73) |  | 44 (10-79) | 5.98 (1.54-10.42) |
| Bangladesh | 141,351 (46,970-247,012) | 96.92 (33.01-168.94) |  | 5,040 (1,813-8,775) | 3.71 (1.39-6.44) |
| Barbados | 1,408 (391-2,528) | 285.40 (76.35-516.19) |  | 63 (20-108) | 12.15 (3.81-21.23) |
| Belarus | 38,874 (9,537-69,663) | 248.39 (60.26-448.53) |  | 1,589 (404-2,809) | 9.85 (2.48-17.45) |
| Belgium | 43,209 (8,500-74,083) | 198.29 (37.95-340.02) |  | 2,216 (457-3,764) | 8.78 (1.78-15.02) |
| Belize | 396 (129-784) | 117.98 (41.69-231.69) |  | 13 (5-26) | 4.40 (1.76-8.52) |
| Benin | 6,177 (2,240-11,443) | 102.58 (43.63-191.40) |  | 204 (88-383) | 3.95 (1.82-7.40) |
| Bermuda | 278 (83-482) | 217.23 (61.03-379.51) |  | 14 (4-23) | 9.51 (2.99-16.24) |
| Bhutan | 594 (119-1,131) | 92.42 (18.93-177.14) |  | 22 (5-42) | 3.68 (0.81-7.03) |
| Bolivia (Plurinational State of) | 22,929 (7,806-51,136) | 241.58 (86.05-538.35) |  | 895 (333-1,993) | 10.38 (4.07-23.05) |
| Bosnia and Herzegovina | 13,923 (3,214-25,185) | 229.72 (52.41-417.31) |  | 623 (150-1,109) | 9.84 (2.36-17.57) |
| Botswana | 3,928 (833-6,885) | 239.31 (54.23-420.02) |  | 136 (31-239) | 9.86 (2.34-17.14) |
| Brazil | 506,192 (107,854-919,978) | 197.86 (42.37-359.41) |  | 19,131 (4,246-34,469) | 7.65 (1.71-13.79) |
| Brunei Darussalam | 1,083 (298-1,894) | 272.19 (79.76-470.10) |  | 36 (11-63) | 11.27 (3.56-19.02) |
| Bulgaria | 45,891 (11,811-80,675) | 349.38 (88.06-616.35) |  | 2,032 (544-3,531) | 14.22 (3.75-24.88) |
| Burkina Faso | 18,723 (4,821-34,090) | 181.18 (51.28-333.42) |  | 652 (193-1,201) | 7.33 (2.39-13.32) |
| Burundi | 9,005 (2,336-15,357) | 157.61 (48.10-261.79) |  | 295 (90-490) | 6.23 (2.32-10.07) |
| Cabo Verde | 1,020 (122-2,143) | 221.60 (28.66-466.64) |  | 42 (7-88) | 9.76 (1.65-20.41) |
| Cambodia | 42,350 (19,513-68,341) | 311.06 (148.28-496.46) |  | 1,464 (715-2,325) | 12.14 (6.25-19.02) |
| Cameroon | 21,482 (5,978-42,321) | 143.78 (47.94-276.69) |  | 695 (236-1,335) | 5.60 (2.15-10.47) |
| Canada | 128,378 (27,168-218,906) | 191.73 (39.44-328.04) |  | 6,196 (1,411-10,498) | 8.26 (1.84-13.96) |
| Central African Republic | 6,909 (1,466-13,227) | 251.84 (60.21-463.65) |  | 210 (48-393) | 9.42 (2.56-17.21) |
| Chad | 10,922 (3,257-21,098) | 167.87 (55.16-321.72) |  | 374 (127-716) | 6.69 (2.58-12.71) |
| Chile | 49,055 (13,653-93,196) | 194.21 (53.72-368.34) |  | 2,213 (642-4,216) | 8.54 (2.47-16.25) |
| China | 4,407,254 (1,479,112-9,106,093) | 210.49 (70.50-431.87) |  | 176,530 (60,802-367,462) | 8.66 (2.98-17.89) |
| Colombia | 91,822 (22,743-187,591) | 166.79 (41.36-340.74) |  | 3,620 (966-7,418) | 6.56 (1.75-13.44) |
| Comoros | 1,282 (321-2,194) | 238.43 (64.93-399.81) |  | 45 (13-75) | 9.47 (2.95-15.23) |
| Congo | 8,427 (2,366-15,433) | 246.59 (82.18-424.86) |  | 257 (83-445) | 8.94 (3.57-15.02) |
| Cook Islands | 33 (7-64) | 138.99 (28.00-270.96) |  | 1 (0-2) | 5.05 (0.81-9.28) |
| Costa Rica | 11,645 (2,980-23,611) | 211.64 (54.21-429.07) |  | 464 (125-946) | 8.44 (2.27-17.20) |
| Côte d'Ivoire | 22,273 (6,181-37,397) | 43.26 (-38.15-89.50) |  | 1,094 (314-1,845) | 0.77 (-3.45-3.18) |
| Croatia | 40,925 (11,005-69,673) | 262.39 (70.95-440.18) |  | 1,838 (544-3,132) | 11.70 (3.31-19.81) |
| Cuba | 3,169 (692-5,483) | 215.13 (56.97-366.97) |  | 147 (36-253) | 9.18 (2.68-15.67) |
| Cyprus | 49,638 (11,707-85,028) | 159.85 (34.62-276.39) |  | 2,359 (579-4,004) | 7.62 (1.87-12.98) |
| Czechia | 8,540 (1,255-16,017) | 243.38 (55.81-419.50) |  | 189 (158-389) | 10.63 (2.56-18.11) |
| Democratic People's Republic of Korea | 92,231 (30,198-192,902) | 270.41 (89.18-561.71) |  | 3,269 (1,109-6,842) | 9.92 (3.39-20.55) |
| Democratic Republic of the Congo | 65,176 (22,069-112,885) | 147.11 (59.20-245.15) |  | 2,060 (816-3,437) | 5.46 (2.62-8.80) |
| Denmark | 26,132 (4,770-44,747) | 228.54 (40.84-392.56) |  | 1,369 (259-2,319) | 10.78 (2.02-18.33) |
| Djibouti | 2,116 (500-4,101) | 280.09 (73.14-522.78) |  | 69 (18-130) | 11.50 (3.36-20.65) |
| Dominica | 198 (36-394) | 237.20 (43.45-474.72) |  | 8 (2-16) | 10.26 (2.06-20.43) |
| Dominican Republic | 13,671 (3,929-25,481) | 132.35 (38.70-245.78) |  | 520 (168-947) | 5.22 (1.70-9.48) |
| Ecuador | 24,670 (6,345-57,310) | 148.18 (38.32-344.59) |  | 997 (272-2,340) | 6.24 (1.73-14.61) |
| Egypt | 89,259 (24,810-169,914) | 120.83 (36.46-224.36) |  | 2,829 (862-5,229) | 4.57 (1.52-8.29) |
| El Salvador | 9,068 (2,201-20,614) | 148.33 (35.88-337.31) |  | 354 (90-816) | 5.63 (1.42-12.97) |
| Equatorial Guinea | 1,279 (270-2,533) | 200.29 (53.16-375.67) |  | 40 (11-74) | 7.60 (2.51-13.65) |
| Eritrea | 10,006 (2,354-18,458) | 299.96 (77.15-541.25) |  | 314 (79-570) | 11.86 (3.42-21.00) |
| Estonia | 5,906 (1,110-10,314) | 231.51 (42.98-407.93) |  | 296 (57-513) | 10.25 (1.96-17.85) |
| Eswatini | 2,435 (502-4,594) | 375.20 (80.70-693.36) |  | 79 (17-145) | 14.31 (3.35-25.66) |
| Ethiopia | 127,448 (44,853-204,053) | 271.15 (102.97-425.94) |  | 4,734 (1,865-7,362) | 11.93 (5.03-18.32) |
| Fiji | 1,774 (432-3,395) | 214.94 (55.53-406.60) |  | 60 (16-114) | 8.58 (2.45-15.76) |
| Finland | 18,579 (3,638-32,029) | 160.39 (30.20-276.88) |  | 948 (196-1,620) | 7.01 (1.41-12.05) |
| France | 282,448 (56,256-481,052) | 221.24 (42.60-381.85) |  | 14,736 (3,048-25,152) | 9.53 (1.97-16.16) |
| Gabon | 2,951 (830-5,345) | 246.49 (80.47-431.34) |  | 98 (33-170) | 9.52 (3.64-16.13) |
| Gambia | 871 (270-1,487) | 79.39 (26.94-131.92) |  | 30 (11-49) | 3.10 (1.21-5.03) |
| Georgia | 13,373 (3,347-24,495) | 238.66 (58.92-437.18) |  | 549 (143-1,004) | 9.27 (2.38-16.97) |
| Germany | 374,131 (65,892-650,108) | 213.77 (36.62-372.73) |  | 18,721 (3,446-32,216) | 9.16 (1.65-15.83) |
| Ghana | 20,022 (5,827-37,395) | 97.28 (25.13-179.40) |  | 615 (165-1,142) | 3.54 (0.57-6.69) |
| Greece | 39,243 (8,622-68,723) | 178.53 (37.40-316.20) |  | 2,112 (491-3,702) | 7.90 (1.78-13.84) |
| Greenland | 256 (48-441) | 351.40 (66.56-607.02) |  | 9 (2-16) | 14.57 (2.80-24.70) |
| Grenada | 234 (88-385) | 201.06 (75.70-330.26) |  | 9 (3-14) | 8.10 (3.26-13.06) |
| Guam | 338 (103-584) | 167.28 (49.97-289.81) |  | 12 (4-20) | 5.63 (1.82-9.50) |
| Guatemala | 16,103 (4,621-42,120) | 137.64 (40.22-359.58) |  | 590 (180-1,566) | 5.48 (1.72-14.63) |
| Guinea | 6,364 (2,184-13,665) | 97.42 (33.92-207.35) |  | 205 (73-435) | 3.53 (1.39-7.46) |
| Guinea-Bissau | 1,968 (557-3,663) | 220.52 (72.44-414.74) |  | 61 (19-114) | 8.43 (3.33-15.63) |
| Guyana | 1,222 (309-2,455) | 175.87 (46.19-349.89) |  | 42 (11-82) | 6.58 (1.93-12.79) |
| Haiti | 19,744 (7,865-37,506) | 228.02 (97.08-434.51) |  | 621 (271-1,188) | 8.24 (3.47-15.38) |
| Honduras | 9,311 (1,958-22,753) | 137.48 (30.51-339.44) |  | 343 (80-849) | 5.64 (1.38-14.03) |
| Hungary | 62,408 (16,549-105,685) | 344.19 (89.78-584.15) |  | 2,800 (769-4,721) | 14.15 (3.84-23.89) |
| Iceland | 947 (179-1,639) | 171.53 (31.92-299.04) |  | 46 (9-78) | 7.49 (1.45-12.86) |
| India | 1,349,872 (475,758-2,348,326) | 105.62 (37.90-183.06) |  | 47,295 (17,521-82,216) | 3.97 (1.51-6.90) |
| Indonesia | 522,304 (243,382-852,416) | 194.81 (97.43-313.42) |  | 17,654 (9,004-28,275) | 7.66 (4.27-12.19) |
| Iran (Islamic Republic of) | 90,945 (24,743-178,657) | 107.98 (30.17-212.32) |  | 3,276 (950-6,411) | 4.36 (1.31-8.57) |
| Iraq | 30,455 (11,100-55,867) | 108.52 (42.67-195.55) |  | 979 (394-1,755) | 4.13 (1.79-7.06) |
| Ireland | 14,954 (2,686-26,353) | 195.46 (34.54-344.62) |  | 701 (135-1,224) | 8.63 (1.64-15.10) |
| Kazakhstan | 32,616 (7,778-63,147) | 171.37 (41.07-329.85) |  | 1,173 (286-2,256) | 6.70 (1.65-12.75) |
| Kenya | 48,670 (3,973-91,040) | 186.25 (16.44-342.27) |  | 1,641 (147-3,019) | 7.47 (0.75-13.62) |
| Kiribati | 226 (68-462) | 269.27 (85.79-539.53) |  | 7 (2-14) | 10.31 (3.61-20.55) |
| Kuwait | 3,484 (839-6,233) | 92.68 (25.65-161.40) |  | 111 (31-195) | 3.88 (1.18-6.68) |
| Kyrgyzstan | 7,950 (1,646-17,851) | 147.74 (31.23-329.84) |  | 265 (57-592) | 5.55 (1.23-12.18) |
| Lao People's Democratic Republic | 12,288 (5,001-20,446) | 231.34 (100.27-385.31) |  | 405 (180-672) | 8.85 (4.31-14.37) |
| Latvia | 9,452 (2,079-16,736) | 260.81 (56.43-464.81) |  | 443 (101-778) | 10.83 (2.43-19.15) |
| Lebanon | 8,122 (2,023-14,609) | 135.64 (33.39-244.98) |  | 351 (99-610) | 5.63 (1.56-9.88) |
| Lesotho | 5,186 (1,470-8,888) | 439.34 (129.18-748.79) |  | 176 (54-298) | 16.69 (5.28-28.00) |
| Liberia | 3,094 (1,157-6,040) | 112.90 (46.33-218.58) |  | 93 (39-182) | 4.05 (1.62-7.72) |
| Libya | 10,483 (3,341-18,831) | 173.22 (58.21-305.96) |  | 354 (121-627) | 6.90 (2.48-12.13) |
| Lithuania | 13,478 (2,794-23,883) | 255.38 (51.66-455.87) |  | 636 (139-1,115) | 10.68 (2.28-18.88) |
| Luxembourg | 1,988 (343-3,370) | 190.18 (32.40-323.53) |  | 98 (18-166) | 8.73 (1.58-14.77) |
| Madagascar | 29,621 (5,518-55,794) | 217.17 (44.11-396.20) |  | 928 (183-1,694) | 8.52 (1.98-14.95) |
| Malawi | 22,217 (646-39,196) | 257.25 (10.79-451.11) |  | 722 (34-1,262) | 9.72 (0.67-16.66) |
| Malaysia | 75,606 (27,946-124,346) | 253.39 (97.57-412.42) |  | 2,765 (1,145-4,423) | 10.14 (4.48-16.02) |
| Maldives | 202 (73-352) | 49.11 (20.36-83.28) |  | 7 (3-12) | 2.05 (1.02-3.38) |
| Mali | 14,938 (2,597-32,603) | 150.35 (28.35-327.57) |  | 507 (98-1,107) | 5.98 (1.29-13.12) |
| Malta | 1,647 (326-2,776) | 183.64 (34.74-315.21) |  | 82 (17-137) | 8.03 (1.63-13.49) |
| Marshall Islands | 112 (41-216) | 261.46 (106.54-494.24) |  | 3 (1-6) | 9.52 (4.27-17.73) |
| Mauritania | 3,925 (815-7,471) | 171.99 (37.44-325.97) |  | 148 (34-279) | 7.28 (1.74-13.66) |
| Mauritius | 3,949 (1,216-7,052) | 216.87 (67.03-386.48) |  | 148 (51-259) | 8.17 (2.88-14.27) |
| Mexico | 180,658 (42,331-350,002) | 136.62 (32.49-265.17) |  | 6,516 (1,659-12,771) | 5.19 (1.35-10.20) |
| Micronesia (Federated States of) | 233 (90-450) | 278.06 (111.49-529.51) |  | 7 (3-14) | 10.49 (4.49-19.55) |
| Monaco | 283 (48-497) | 328.14 (52.28-582.03) |  | 14 (3-25) | 14.00 (2.46-24.49) |
| Mongolia | 9,317 (2,079-19,807) | 366.93 (79.85-772.85) |  | 326 (70-687) | 15.07 (3.11-31.60) |
| Montenegro | 2,134 (438-3,719) | 223.73 (45.33-390.83) |  | 93 (21-163) | 9.88 (2.17-17.27) |
| Morocco | 38,647 (11,905-69,063) | 106.19 (33.48-186.60) |  | 1,385 (462-2,400) | 4.15 (1.44-7.12) |
| Mozambique | 19,583 (3,314-36,382) | 159.10 (31.13-289.51) |  | 673 (133-1,218) | 6.68 (1.54-11.87) |
| Myanmar | 117,523 (45,312-202,398) | 224.69 (89.63-383.58) |  | 4,110 (1,717-6,934) | 8.62 (3.76-14.45) |
| Namibia | 2,736 (666-5,321) | 171.46 (44.68-327.35) |  | 88 (24-167) | 6.35 (1.87-11.68) |
| Nauru | 23 (7-46) | 339.51 (107.75-644.12) |  | 1 (0-1) | 12.55 (4.45-23.71) |
| Nepal | 26,010 (5,017-50,318) | 104.81 (20.81-202.19) |  | 914 (187-1,772) | 4.04 (0.86-7.80) |
| Netherlands | 88,101 (15,689-149,802) | 264.36 (46.26-450.73) |  | 4,231 (782-7,205) | 11.54 (2.11-19.69) |
| New Zealand | 18,669 (4,718-31,138) | 233.10 (57.48-390.11) |  | 888 (233-1,448) | 10.22 (2.66-16.75) |
| Nicaragua | 5,472 (1,786-11,113) | 104.11 (34.85-212.29) |  | 197 (68-406) | 4.09 (1.45-8.45) |
| Niger | 10,194 (2,201-22,035) | 113.36 (27.32-244.74) |  | 358 (87-778) | 4.72 (1.34-10.03) |
| Nigeria | 102,524 (15,923-190,331) | 80.50 (-10.55-150.48) |  | 2,722 (587-5,084) | 2.15 (-2.90-4.94) |
| Niue | 5 (1-9) | 224.37 (69.67-426.21) |  | 0 (0-0) | 8.92 (3.14-16.49) |
| North Macedonia | 7,942 (2,036-14,825) | 241.83 (62.01-448.40) |  | 335 (89-630) | 10.95 (2.94-20.30) |
| Northern Mariana Islands | 129 (38-234) | 232.31 (71.44-417.90) |  | 4 (1-8) | 9.58 (3.30-16.91) |
| Norway | 20,273 (3,698-33,518) | 208.61 (37.30-345.85) |  | 1,060 (206-1,743) | 9.80 (1.86-16.13) |
| Oman | 1,184 (232-2,428) | 49.96 (10.49-101.27) |  | 39 (8-80) | 2.16 (0.49-4.34) |
| Pakistan | 274,395 (40,470-527,017) | 190.09 (30.26-359.27) |  | 8,783 (1,437-16,513) | 7.15 (1.31-13.17) |
| Palau | 58 (16-112) | 254.76 (73.85-482.64) |  | 2 (1-4) | 10.90 (3.71-20.19) |
| Palestine | 5,949 (2,586-9,426) | 209.55 (97.24-329.07) |  | 205 (97-319) | 8.80 (4.38-13.27) |
| Panama | 6,408 (2,162-12,205) | 145.07 (48.94-276.34) |  | 247 (92-474) | 5.55 (2.07-10.66) |
| Papua New Guinea | 8,204 (2,294-17,853) | 119.71 (38.81-268.43) |  | 231 (74-517) | 3.97 (1.24-9.42) |
| Paraguay | 10,513 (2,123-19,839) | 173.33 (35.80-327.53) |  | 404 (89-754) | 7.07 (1.61-13.19) |
| Peru | 47,469 (12,774-114,691) | 138.11 (37.82-334.22) |  | 1,916 (587-4,723) | 5.72 (1.77-14.14) |
| Philippines | 217,108 (101,814-335,907) | 232.47 (114.81-351.92) |  | 6,997 (3,609-10,432) | 8.36 (4.61-12.26) |
| Poland | 203,100 (56,637-344,516) | 293.46 (80.56-499.69) |  | 9,708 (2,802-16,308) | 13.13 (3.76-22.16) |
| Portugal | 47,721 (11,629-84,633) | 216.40 (51.22-386.28) |  | 2,427 (613-4,263) | 9.24 (2.27-16.31) |
| Puerto Rico | 10,838 (2,785-18,590) | 177.32 (43.62-309.66) |  | 499 (138-852) | 6.87 (1.79-11.83) |
| Qatar | 1,474 (314-2,778) | 116.83 (27.90-210.69) |  | 41 (10-77) | 5.44 (1.35-9.59) |
| Republic of Korea | 140,430 (41,480-266,731) | 154.61 (45.51-293.97) |  | 6,547 (2,050-12,280) | 7.07 (2.22-13.26) |
| Republic of Moldova | 15,204 (3,910-26,372) | 257.97 (65.85-449.37) |  | 597 (159-1,027) | 9.93 (2.63-17.15) |
| Romania | 95,228 (21,648-173,482) | 276.32 (62.00-502.50) |  | 4,170 (974-7,598) | 11.12 (2.57-20.23) |
| Russian Federation | 652,082 (165,364-1,162,795) | 278.91 (70.64-498.98) |  | 27,739 (7,340-49,014) | 11.54 (3.06-20.43) |
| Rwanda | 14,040 (1,914-25,748) | 196.39 (30.89-353.63) |  | 475 (77-860) | 7.98 (1.51-14.16) |
| Saint Kitts and Nevis | 159 (50-281) | 217.68 (73.52-379.86) |  | 6 (2-10) | 9.23 (3.69-15.63) |
| Saint Lucia | 362 (110-672) | 150.01 (44.01-278.90) |  | 13 (4-24) | 5.53 (1.62-10.20) |
| Saint Vincent and the Grenadines | 245 (82-447) | 173.72 (57.48-317.19) |  | 9 (3-16) | 6.39 (1.96-11.31) |
| Samoa | 240 (77-457) | 157.21 (52.85-294.78) |  | 9 (3-16) | 6.20 (2.38-11.47) |
| San Marino | 100 (23-200) | 144.39 (31.90-298.68) |  | 5 (1-11) | 6.35 (1.52-12.68) |
| Sao Tome and Principe | 182 (74-353) | 149.87 (69.13-286.60) |  | 7 (3-13) | 6.37 (3.51-12.07) |
| Saudi Arabia | 28,703 (6,637-52,201) | 100.30 (25.87-175.67) |  | 791 (196-1,408) | 3.83 (1.07-6.54) |
| Senegal | 12,452 (4,093-23,317) | 145.60 (54.39-271.96) |  | 437 (175-811) | 5.79 (2.51-10.76) |
| Serbia | 43,354 (9,982-76,045) | 275.91 (61.80-485.23) |  | 1,939 (471-3,373) | 11.58 (2.76-20.19) |
| Seychelles | 250 (115-382) | 199.33 (94.42-297.38) |  | 9 (4-13) | 7.43 (3.52-10.73) |
| Sierra Leone | 4,745 (1,964-8,736) | 107.08 (47.64-196.89) |  | 155 (72-286) | 3.98 (1.90-7.51) |
| Singapore | 12,048 (3,289-20,925) | 139.65 (38.54-242.36) |  | 516 (156-879) | 6.11 (1.87-10.41) |
| Slovakia | 29,747 (7,534-50,183) | 319.64 (79.95-538.61) |  | 1,300 (340-2,193) | 13.54 (3.51-22.87) |
| Slovenia | 8,350 (1,850-15,129) | 196.33 (42.55-355.23) |  | 433 (100-793) | 9.05 (2.07-16.58) |
| Solomon Islands | 857 (304-1,819) | 195.03 (74.64-401.72) |  | 25 (10-52) | 6.74 (2.71-13.77) |
| Somalia | 22,010 (5,789-40,177) | 298.83 (84.87-529.74) |  | 686 (191-1,226) | 11.60 (3.61-19.87) |
| South Africa | 139,446 (36,460-230,517) | 279.88 (74.94-460.73) |  | 4,985 (1,388-8,206) | 11.15 (3.21-18.36) |
| South Sudan | 11,658 (2,060-21,745) | 258.00 (50.79-470.33) |  | 383 (74-698) | 10.20 (2.33-18.16) |
| Spain | 182,540 (41,646-311,425) | 199.80 (43.79-342.80) |  | 9,280 (2,199-15,670) | 8.68 (2.02-14.73) |
| Sri Lanka | 23,643 (7,199-43,337) | 86.31 (26.74-157.62) |  | 910 (302-1,614) | 3.41 (1.17-6.01) |
| Sudan | 30,685 (7,565-66,623) | 132.50 (35.02-284.01) |  | 1,003 (274-2,146) | 5.12 (1.50-10.74) |
| Suriname | 1,135 (430-1,953) | 174.59 (67.54-299.65) |  | 42 (18-71) | 6.74 (2.93-11.25) |
| Sweden | 34,739 (6,921-59,440) | 169.86 (32.85-292.28) |  | 1,895 (392-3,201) | 7.97 (1.62-13.55) |
| Switzerland | 24,771 (4,787-42,594) | 144.07 (27.16-248.17) |  | 1,268 (253-2,175) | 6.45 (1.27-11.07) |
| Syrian Arab Republic | 13,388 (3,653-26,370) | 94.96 (26.79-184.68) |  | 460 (135-890) | 3.70 (1.15-7.01) |
| Taiwan (Province of China) | 109,555 (30,389-182,110) | 270.22 (73.74-451.43) |  | 4,713 (1,422-7,834) | 11.11 (3.34-18.51) |
| Tajikistan | 8,897 (2,194-18,623) | 130.94 (32.60-277.78) |  | 286 (72-610) | 5.04 (1.24-10.76) |
| Thailand | 254,879 (92,549-442,315) | 243.94 (88.25-424.85) |  | 9,756 (3,970-16,411) | 9.09 (3.70-15.24) |
| Timor-Leste | 1,900 (877-3,065) | 211.19 (100.13-336.34) |  | 69 (34-110) | 8.29 (4.24-12.88) |
| Togo | 7,491 (2,828-13,989) | 163.80 (72.74-296.26) |  | 241 (106-437) | 6.28 (3.09-11.13) |
| Tokelau | 3 (1-5) | 203.64 (70.37-375.39) |  | 0 (0-0) | 7.61 (2.92-13.47) |
| Tonga | 163 (46-324) | 196.02 (56.98-390.62) |  | 6 (2-12) | 7.44 (2.23-14.58) |
| Trinidad and Tobago | 3,247 (1,114-5,503) | 171.19 (58.98-291.83) |  | 114 (40-182) | 5.93 (2.04-9.43) |
| Tunisia | 13,425 (3,164-25,758) | 98.23 (23.47-187.62) |  | 506 (130-951) | 3.92 (1.02-7.31) |
| Türkiye | 5,305 (1,011-11,409) | 146.04 (37.72-278.55) |  | 170 (33-366) | 5.95 (1.66-11.19) |
| Turkmenistan | 25 (10-47) | 116.92 (22.58-251.20) |  | 1 (0-2) | 4.17 (0.83-8.92) |
| Tuvalu | 139,412 (35,610-266,492) | 230.90 (91.66-426.14) |  | 5,411 (1,484-10,263) | 8.79 (3.97-16.04) |
| Uganda | 44,583 (5,377-83,347) | 259.40 (35.53-480.08) |  | 1,470 (209-2,708) | 10.16 (1.66-18.37) |
| Ukraine | 178,595 (46,402-340,622) | 242.13 (62.41-465.90) |  | 7,064 (1,882-13,383) | 9.11 (2.41-17.34) |
| United Arab Emirates | 6,675 (1,421-12,391) | 142.82 (34.74-254.60) |  | 188 (42-347) | 6.79 (1.77-12.08) |
| United Kingdom | 284,356 (54,342-486,728) | 233.49 (43.28-399.77) |  | 14,630 (2,965-24,813) | 10.57 (2.10-17.97) |
| United Republic of Tanzania | 63,009 (11,886-114,448) | 218.73 (44.93-389.35) |  | 2,175 (466-3,855) | 8.87 (2.11-15.37) |
| United States of America | 1,082,048 (202,381-1,844,641) | 201.95 (37.03-345.63) |  | 46,954 (9,398-78,941) | 8.02 (1.58-13.52) |
| United States Virgin Islands | 285 (72-526) | 183.04 (43.58-341.82) |  | 12 (3-23) | 7.26 (1.97-13.15) |
| Uruguay | 19,038 (4,203-33,040) | 365.76 (79.10-636.98) |  | 941 (218-1,622) | 16.07 (3.66-27.75) |
| Uzbekistan | 30,554 (6,007-64,732) | 99.53 (20.11-208.31) |  | 954 (198-1,993) | 3.50 (0.76-7.36) |
| Vanuatu | 424 (145-850) | 209.80 (78.92-411.98) |  | 13 (5-26) | 7.94 (3.48-15.00) |
| Venezuela (Bolivarian Republic of) | 50,447 (15,374-97,616) | 163.69 (50.97-316.27) |  | 1,820 (605-3,515) | 6.11 (2.08-11.80) |
| Viet Nam | 223,541 (97,433-369,420) | 210.01 (93.62-344.17) |  | 8,055 (3,743-13,052) | 8.25 (3.94-13.25) |
| Yemen | 25,138 (9,289-56,726) | 152.08 (58.77-340.52) |  | 829 (325-1,862) | 5.92 (2.54-13.17) |
| Zambia | 32,281 (10,705-60,994) | 372.15 (138.98-677.74) |  | 997 (371-1,818) | 14.08 (5.94-24.40) |
| Zimbabwe | 34,086 (10,784-58,635) | 418.66 (143.09-700.07) |  | 1,096 (374-1,836) | 15.96 (5.93-25.52) |
| Abbreviations: ASR, age standardized rate; UI, uncertainty interval. | | | | | |

| **Table S5.** Prediction of ASDR and ASMR of DRNs in global from 2022 to 2050. | | | | | | | | | | | | | | | |
| --- | --- | --- | --- | --- | --- | --- | --- | --- | --- | --- | --- | --- | --- | --- | --- |
| **Year** |  | **Predicted ASDR** | | | | | |  | **Predicted ASMR** | | | | | | |
|  | **DRNs** | **EC** | **SC** | **CRC** | **TBLC** | **BC** | **PC** |  | **DRNs** | **EC** | **SC** | **CRC** | **TBLC** | **BC** | **PC** |
| 2022 | 187.53 (184.20 – 190.86) | 15.51 (14.80 – 16.22) | 20.36 (19.81 – 20.91) | 109.26 (107.18 – 111.35) | 18.29 (17.82 – 18.77) | 28.03 (27.55 – 28.50) | -3.73 (-3.63 – -0.83) |  | 7.82 (7.68 – 7.95) | 0.65 (0.63 – 0.67) | 0.87 (0.85 – 0.89) | 4.80 (4.72 – 4.89) | 0.76 (0.75 – 0.78) | 0.95 (0.93 – 0.97) | -0.20 (-0.19 – -0.20) |
| 2023 | 186.01 (180.62 – 191.39) | 15.42 (14.48 – 16.35) | 20.01 (19.16 – 20.87) | 108.42 (105.23 – 111.61) | 18.12 (17.44 – 18.80) | 27.94 (27.19 – 28.70) | -3.74 (-3.60 – -3.87) |  | 7.74 (7.53 – 7.96) | 0.65 (0.61 – 0.68) | 0.86 (0.82 – 0.89) | 4.76 (4.63 – 4.89) | 0.75 (0.73 – 0.78) | 0.95 (0.92 – 0.97) | -0.20 (-0.19 – -0.21) |
| 2024 | 184.50 (176.54 – 192.46) | 15.33 (14.09 – 16.56) | 19.67 (18.43 – 20.91) | 107.57 (102.98 – 112.17) | 17.94 (17.00 – 18.89) | 27.88 (26.78 – 28.98) | -3.75 (-3.56 – -3.93) |  | 7.67 (7.35 – 7.99) | 0.64 (0.60 – 0.69) | 0.85 (0.79 – 0.90) | 4.72 (4.53 – 4.91) | 0.75 (0.71 – 0.78) | 0.94 (0.91 – 0.98) | -0.20 (-0.19 – -0.21) |
| 2025 | 182.97 (172.07 – 193.87) | 15.25 (13.66 – 16.84) | 19.31 (17.64 – 20.99) | 106.71 (100.51 – 112.91) | 17.76 (16.51 – 19.01) | 27.82 (26.31 – 29.32) | -3.76 (-3.52 – -4.00) |  | 7.60 (7.16 – 8.04) | 0.64 (0.58 – 0.70) | 0.83 (0.76 – 0.90) | 4.68 (4.42 – 4.94) | 0.74 (0.69 – 0.79) | 0.94 (0.89 – 0.99) | -0.20 (-0.19 – -0.21) |
| 2026 | 181.45 (167.31 – 195.59) | 15.18 (13.20 – 17.16) | 18.97 (16.83 – 21.11) | 105.82 (97.85 – 113.80) | 17.58 (15.99 – 19.17) | 27.76 (25.81 – 29.71) | -3.77 (-3.46 – -4.07) |  | 7.53 (6.96 – 8.10) | 0.63 (0.56 – 0.71) | 0.82 (0.73 – 0.91) | 4.64 (4.31 – 4.97) | 0.73 (0.67 – 0.80) | 0.94 (0.87 – 1.00) | -0.20 (-0.18 – -0.22) |
| 2027 | 179.98 (162.33 – 197.64) | 15.12 (12.70 – 17.53) | 18.63 (15.99 – 21.27) | 104.95 (95.06 – 114.85) | 17.39 (15.44 – 19.35) | 27.72 (25.28 – 30.16) | -3.77 (-3.40 – -4.15) |  | 7.46 (6.75 – 8.17) | 0.63 (0.53 – 0.73) | 0.81 (0.69 – 0.92) | 4.60 (4.19 – 5.01) | 0.73 (0.65 – 0.80) | 0.94 (0.85 – 1.02) | -0.20 (-0.18 – -0.22) |
| 2028 | 178.62 (157.23 – 200.01) | 15.07 (12.18 – 17.95) | 18.30 (15.14 – 21.47) | 104.12 (92.18 – 116.06) | 17.21 (14.88 – 19.55) | 27.71 (24.74 – 30.69) | -3.78 (-3.33 – -4.23) |  | 7.40 (6.54 – 8.25) | 0.63 (0.51 – 0.74) | 0.79 (0.66 – 0.93) | 4.56 (4.06 – 5.06) | 0.72 (0.62 – 0.81) | 0.93 (0.83 – 1.03) | -0.20 (-0.17 – -0.23) |
| 2029 | 177.29 (151.97 – 202.62) | 15.02 (11.64 – 18.40) | 17.98 (14.28 – 21.68) | 103.30 (89.22 – 117.38) | 17.03 (14.29 – 19.76) | 27.72 (24.18 – 31.27) | -3.79 (-3.25 – -4.32) |  | 7.33 (6.32 – 8.35) | 0.62 (0.49 – 0.76) | 0.78 (0.62 – 0.94) | 4.52 (3.93 – 5.11) | 0.71 (0.60 – 0.82) | 0.93 (0.81 – 1.05) | -0.20 (-0.17 – -0.23) |
| 2030 | 175.93 (146.51 – 205.35) | 14.99 (11.08 – 18.89) | 17.64 (13.40 – 21.89) | 102.45 (86.14 – 118.75) | 16.84 (13.69 – 19.99) | 27.74 (23.60 – 31.89) | -3.80 (-3.18 – -4.42) |  | 7.27 (6.09 – 8.44) | 0.62 (0.46 – 0.78) | 0.77 (0.58 – 0.95) | 4.48 (3.80 – 5.16) | 0.70 (0.58 – 0.83) | 0.93 (0.79 – 1.07) | -0.20 (-0.16 – -0.23) |
| 2031 | 174.57 (140.90 – 208.24) | 14.96 (10.51 – 19.41) | 17.32 (12.52 – 22.12) | 101.57 (82.95 – 120.18) | 16.66 (13.08 – 20.23) | 27.77 (22.99 – 32.55) | -3.81 (-3.09 – -4.52) |  | 7.20 (5.86 – 8.55) | 0.62 (0.44 – 0.80) | 0.75 (0.54 – 0.96) | 4.44 (3.67 – 5.22) | 0.69 (0.55 – 0.84) | 0.93 (0.77 – 1.09) | -0.20 (-0.16 – -0.24) |
| 2032 | 173.27 (135.18 – 211.35) | 14.94 (9.91 – 19.96) | 17.01 (11.64 – 22.38) | 100.70 (79.70 – 121.69) | 16.47 (12.45 – 20.49) | 27.81 (22.36 – 33.26) | -3.82 (-3.00 – -4.63) |  | 7.14 (5.62 – 8.66) | 0.62 (0.41 – 0.82) | 0.74 (0.51 – 0.98) | 4.40 (3.53 – 5.28) | 0.69 (0.52 – 0.85) | 0.93 (0.75 – 1.11) | -0.20 (-0.15 – -0.25) |
| 2033 | 172.07 (129.42 – 214.73) | 14.92 (9.29 – 20.55) | 16.71 (10.76 – 22.65) | 99.88 (76.42 – 123.34) | 16.29 (11.82 – 20.76) | 27.88 (21.72 – 34.04) | -3.82 (-2.91 – -4.74) |  | 7.08 (5.38 – 8.78) | 0.61 (0.39 – 0.84) | 0.73 (0.47 – 0.99) | 4.37 (3.39 – 5.34) | 0.68 (0.50 – 0.86) | 0.93 (0.72 – 1.14) | -0.20 (-0.15 – -0.25) |
| 2034 | 170.92 (123.57 – 218.27) | 14.92 (8.66 – 21.18) | 16.41 (9.88 – 22.93) | 99.07 (73.10 – 125.05) | 16.11 (11.19 – 21.04) | 27.97 (21.07 – 34.88) | -3.84 (-2.82 – -4.85) |  | 7.02 (5.14 – 8.91) | 0.61 (0.36 – 0.87) | 0.72 (0.43 – 1.00) | 4.33 (3.25 – 5.41) | 0.67 (0.47 – 0.87) | 0.93 (0.70 – 1.16) | -0.20 (-0.14 – -0.26) |
| 2035 | 169.72 (117.58 – 221.87) | 14.92 (8.01 – 21.83) | 16.11 (9.01 – 23.21) | 98.24 (69.70 – 126.77) | 15.93 (10.54 – 21.32) | 28.08 (20.39 – 35.76) | -3.85 (-2.72 – -4.98) |  | 6.97 (4.89 – 9.04) | 0.61 (0.33 – 0.89) | 0.71 (0.39 – 1.02) | 4.29 (3.10 – 5.48) | 0.66 (0.45 – 0.88) | 0.93 (0.68 – 1.19) | -0.20 (-0.14 – -0.26) |
| 2036 | 168.52 (111.48 – 225.56) | 14.92 (7.34 – 22.51) | 15.81 (8.13 – 23.48) | 97.36 (66.23 – 128.49) | 15.75 (9.89 – 21.62) | 28.19 (19.69 – 36.69) | -3.86 (-2.61 – -5.10) |  | 6.91 (4.64 – 9.18) | 0.61 (0.30 – 0.92) | 0.69 (0.36 – 1.03) | 4.25 (2.96 – 5.55) | 0.65 (0.42 – 0.89) | 0.93 (0.65 – 1.21) | -0.20 (-0.13 – -0.27) |
| 2037 | 167.37 (105.31 – 229.42) | 14.94 (6.65 – 23.22) | 15.52 (7.27 – 23.77) | 96.49 (62.72 – 130.27) | 15.58 (9.24 – 21.92) | 28.32 (18.96 – 37.67) | -3.87 (-2.50 – -5.23) |  | 6.86 (4.39 – 9.32) | 0.61 (0.27 – 0.94) | 0.68 (0.32 – 1.05) | 4.22 (2.81 – 5.62) | 0.65 (0.39 – 0.90) | 0.93 (0.63 – 1.24) | -0.20 (-0.12 – -0.28) |
| 2038 | 166.32 (99.12 – 233.51) | 14.95 (5.94 – 23.97) | 15.25 (6.42 – 24.08) | 95.68 (59.21 – 132.16) | 15.41 (8.58 – 22.23) | 28.47 (18.21 – 38.73) | -3.88 (-2.39 – -5.36) |  | 6.80 (4.14 – 9.47) | 0.61 (0.24 – 0.97) | 0.67 (0.28 – 1.06) | 4.18 (2.66 – 5.70) | 0.64 (0.37 – 0.91) | 0.94 (0.60 – 1.27) | -0.20 (-0.12 – -0.28) |
| 2039 | 165.29 (92.86 – 237.73) | 14.98 (5.21 – 24.74) | 14.97 (5.57 – 24.38) | 94.88 (55.67 – 134.09) | 15.24 (7.93 – 22.55) | 28.65 (17.45 – 39.85) | -3.89 (-2.28 – -5.50) |  | 6.75 (3.89 – 9.62) | 0.61 (0.21 – 1.00) | 0.66 (0.24 – 1.08) | 4.14 (2.51 – 5.77) | 0.63 (0.34 – 0.92) | 0.94 (0.57 – 1.30) | -0.20 (-0.11 – -0.29) |
| 2040 | 164.23 (86.49 – 241.96) | 15.00 (4.46 – 25.54) | 14.70 (4.73 – 24.66) | 94.05 (52.09 – 136.00) | 15.07 (7.27 – 22.87) | 28.84 (16.65 – 41.03) | -3.90 (-2.16 – -5.64) |  | 6.70 (3.63 – 9.77) | 0.61 (0.18 – 1.03) | 0.65 (0.21 – 1.09) | 4.11 (2.36 – 5.85) | 0.62 (0.31 – 0.94) | 0.94 (0.54 – 1.34) | -0.20 (-0.10 – -0.30) |
| 2041 | 163.14 (80.05 – 246.24) | 15.03 (3.69 – 26.36) | 14.42 (3.90 – 24.94) | 93.17 (48.47 – 137.87) | 14.91 (6.62 – 23.20) | 29.04 (15.81 – 42.26) | -3.91 (-2.04 – -5.79) |  | 6.65 (3.37 – 9.93) | 0.61 (0.15 – 1.07) | 0.64 (0.17 – 1.10) | 4.07 (2.21 – 5.93) | 0.62 (0.28 – 0.95) | 0.94 (0.52 – 1.37) | -0.20 (-0.10 – -0.31) |
| 2042 | 162.09 (73.55 – 250.64) | 15.06 (2.90 – 27.22) | 14.16 (3.08 – 25.23) | 92.30 (44.82 – 139.77) | 14.75 (5.96 – 23.54) | 29.26 (14.95 – 43.57) | -3.92 (-1.91 – -5.94) |  | 6.60 (3.11 – 10.09) | 0.61 (0.12 – 1.10) | 0.63 (0.13 – 1.12) | 4.03 (2.06 – 6.01) | 0.61 (0.26 – 0.96) | 0.95 (0.49 – 1.41) | -0.20 (-0.09 – -0.31) |
| 2043 | 161.14 (67.03 – 255.25) | 15.10 (2.09 – 28.11) | 13.90 (2.28 – 25.53) | 91.49 (41.19 – 141.78) | 14.59 (5.30 – 23.89) | 29.50 (14.05 – 44.95) | -3.94 (-1.78 – -6.10) |  | 6.55 (2.85 – 10.26) | 0.61 (0.09 – 1.13) | 0.62 (0.10 – 1.13) | 4.00 (1.91 – 6.09) | 0.60 (0.23 – 0.97) | 0.95 (0.46 – 1.45) | -0.20 (-0.08 – -0.32) |
| 2044 | 160.21 (60.45 – 259.98) | 15.14 (1.25 – 29.02) | 13.65 (1.48 – 25.83) | 90.70 (37.56 – 143.84) | 14.45 (4.65 – 24.24) | 29.76 (13.12 – 46.41) | -3.95 (-1.64 – -6.26) |  | 6.50 (2.58 – 10.43) | 0.61 (0.05 – 1.17) | 0.61 (0.06 – 1.15) | 3.96 (1.75 – 6.17) | 0.59 (0.20 – 0.98) | 0.96 (0.43 – 1.49) | -0.20 (-0.08 – -0.33) |
| 2045 | 159.25 (53.80 – 264.69) | 15.18 (0.40 – 29.97) | 13.40 (0.70 – 26.10) | 89.87 (33.90 – 145.84) | 14.30 (3.99 – 24.61) | 30.04 (12.15 – 47.93) | -3.97 (-1.51 – -6.43) |  | 6.46 (2.32 – 10.60) | 0.61 (0.02 – 1.21) | 0.60 (0.03 – 1.16) | 3.92 (1.60 – 6.25) | 0.59 (0.18 – 1.00) | 0.96 (0.39 – 1.53) | -0.20 (-0.07 – -0.34) |
| 2046 | 158.25 (47.09 – 269.41) | 15.23 (-0.48 – 30.94) | 13.14 (-0.07 – 26.36) | 88.99 (30.21 – 147.77) | 14.16 (3.34 – 24.97) | 30.33 (11.13 – 49.54) | -3.99 (-1.37 – -6.60) |  | 6.41 (2.05 – 10.77) | 0.61 (-0.02 – 1.25) | 0.59 (-0.01 – 1.18) | 3.89 (1.45 – 6.33) | 0.58 (0.15 – 1.01) | 0.97 (0.36 – 1.57) | -0.20 (-0.06 – -0.35) |
| 2047 | 157.28 (40.33 – 274.22) | 15.28 (-1.39 – 31.94) | 12.90 (-0.82 – 26.63) | 88.11 (26.52 – 149.70) | 14.02 (2.69 – 25.34) | 30.64 (10.06 – 51.22) | -4.00 (-1.22 – -6.78) |  | 6.37 (1.78 – 10.95) | 0.62 (-0.06 – 1.29) | 0.58 (-0.04 – 1.19) | 3.85 (1.29 – 6.41) | 0.57 (0.12 – 1.02) | 0.97 (0.33 – 1.62) | -0.20 (-0.05 – -0.36) |
| 2048 | 156.38 (33.54 – 279.21) | 15.33 (-2.31 – 32.97) | 12.67 (-1.57 – 26.90) | 87.29 (22.85 – 151.73) | 13.88 (2.04 – 25.73) | 30.97 (8.94 – 53.00) | -4.02 (-1.07 – -6.97) |  | 6.33 (1.52 – 11.13) | 0.62 (-0.09 – 1.33) | 0.57 (-0.07 – 1.21) | 3.82 (1.14 – 6.49) | 0.57 (0.10 – 1.03) | 0.98 (0.29 – 1.67) | -0.20 (-0.04 – -0.37) |
| 2049 | 155.50 (26.71 – 284.29) | 15.38 (-3.27 – 34.02) | 12.43 (-2.30 – 27.16) | 86.49 (19.19 – 153.79) | 13.76 (1.39 – 26.12) | 31.32 (7.77 – 54.87) | -4.04 (-0.92 – -7.16) |  | 6.28 (1.25 – 11.32) | 0.62 (-0.13 – 1.37) | 0.56 (-0.11 – 1.22) | 3.78 (0.98 – 6.57) | 0.56 (0.07 – 1.05) | 0.99 (0.26 – 1.72) | -0.21 (-0.04 – -0.37) |
| 2050 | 154.59 (19.83 – 289.35) | 15.43 (-4.24 – 35.11) | 12.19 (-3.01 – 27.40) | 85.66 (15.52 – 155.79) | 13.63 (0.74 – 26.53) | 31.68 (6.54 – 56.82) | -4.06 (-0.77 – -7.36) |  | 6.24 (0.98 – 11.51) | 0.62 (-0.17 – 1.41) | 0.55 (-0.14 – 1.24) | 3.74 (0.83 – 6.66) | 0.55 (0.05 – 1.06) | 1.00 (0.22 – 1.77) | -0.21 (-0.03 – -0.38) |
| Abbreviations:ASDR, age-standardized rates of DALYs; ASMR, age-standardized mortality rates; DRNs, diet-related neoplasms; EC, Colon and rectum cancer; SC, Stomach cancer; CRC, Colon and rectum cancer; TBLC, Tracheal, bronchus, and lung cancer; BC, Breast cancer; PC, prostate cancer. | | | | | | | | | | | | | | | |

| **Table S6.** Summary measures for SDI-related inequalities in DALYs of DRNs. | | | | | |
| --- | --- | --- | --- | --- | --- |
| **Diseases** | **Health inequality metrics** | **Year** | **Value** | **upper** | **lower** |
| **DRNs** | Slope index of inequality | 1990 | 262.74 | 314.10 | 211.38 |
|  |  | 2021 | 296.14 | 337.11 | 255.17 |
|  | Concentration index | 1990 | 0.27 | 2.07 | -0.09 |
|  |  | 2021 | 0.30 | 2.21 | -0.21 |
| **EC** | Slope index of inequality | 1990 | -2.63 | 3.93 | -9.19 |
|  |  | 2021 | -0.90 | 4.93 | -6.72 |
|  | Concentration index | 1990 | 0.02 | 2.42 | -0.35 |
|  |  | 2021 | 0.01 | 1.95 | 0.06 |
| **SC** | Slope index of inequality | 1990 | 20.55 | 26.00 | 15.11 |
|  |  | 2021 | 11.20 | 15.50 | 6.90 |
|  | Concentration index | 1990 | 0.17 | 2.17 | -0.22 |
|  |  | 2021 | 0.28 | 2.62 | -0.54 |
| **CRC** | Slope index of inequality | 1990 | 156.66 | 187.46 | 125.86 |
|  |  | 2021 | 213.91 | 241.65 | 186.17 |
|  | Concentration index | 1990 | 0.37 | 2.25 | -0.30 |
|  |  | 2021 | 0.37 | 2.38 | -0.40 |
| **TBLC** | Slope index of inequality | 1990 | 16.22 | 21.21 | 11.24 |
|  |  | 2021 | 14.27 | 18.83 | 9.71 |
|  | Concentration index | 1990 | 0.18 | 1.92 | -0.01 |
|  |  | 2021 | 0.17 | 1.95 | 0.06 |
| **BC** | Slope index of inequality | 1990 | 51.74 | 60.19 | 43.30 |
|  |  | 2021 | 48.25 | 55.63 | 40.88 |
|  | Concentration index | 1990 | 0.41 | 2.47 | -0.49 |
|  |  | 2021 | 0.24 | 2.06 | -0.02 |
| **PC** | Slope index of inequality | 1990 | 4.12 | 5.43 | 2.81 |
|  |  | 2021 | 4.73 | 6.58 | 2.89 |
|  | Concentration index | 1990 | -0.23 | 2.15 | -0.22 |
|  |  | 2021 | -0.14 | 2.14 | -0.16 |
| Abbreviations: SDI, sociodemographic index; DALYs, disability-adjusted life years; DRNs, diet-related neoplasms; EC, Colon and rectum cancer; SC, Stomach cancer; CRC, Colon and rectum cancer; TBLC, Tracheal, bronchus, and lung cancer; BC, Breast cancer; PC, Prostate cancer. | | | | | |
